# Evaluating the ability of some natural phenolic acids to target the main protease and AAK1 in SARS COV-2

**DOI:** 10.1038/s41598-023-34189-6

**Published:** 2023-05-05

**Authors:** Heba I. Ghamry, Amany Belal, Mohamed Kandeel El-Ashrey, Haytham O. Tawfik, Reem I. Alsantali, Ahmad J. Obaidullah, Ahmed A. El-Mansi, Doaa Abdelrahman

**Affiliations:** 1 Nutrition and Food Sciences, Department of Home Economics, College of Home Economics, King Khalid University, P.O. Box 960, Abha, 61421 Saudi Arabia; 2 Department of Pharmaceutical Chemistry, College of Pharmacy, Taif University, P.O. Box 11099, Taif, 21944 Saudi Arabia; 3 Pharmaceutical Chemistry Department, Faculty of Pharmacy, Cairo University, Kasr Elini St., Cairo, 11562 Egypt; 4 Medicinal Chemistry Department, Faculty of Pharmacy, King Salman International University, Ras-Sedr, South Sinai Egypt; 5 Department of Pharmaceutical Chemistry, Faculty of Pharmacy, Tanta University, Tanta, 31527 Egypt; 6 Department of Pharmaceutical Chemistry, College of Pharmacy, King Saud University, P.O. Box 2457, Riyadh, 11451 Saudi Arabia; 7 Biology Department, Faculty of Science, King Khalid University, Abha, Saudi Arabia; 8 Zoology Department, Faculty of Science, Mansoura University, Mansoura, Egypt; 9 Department of Clinical Sciences, College of Medicine, Princess Nourah bint Abdulrahman University, Riyadh, Saudi Arabia

**Keywords:** Medicinal chemistry, Theoretical chemistry, Virtual drug screening, Target identification

## Abstract

Researchers are constantly searching for drugs to combat the coronavirus pandemic caused by SARS-CoV-2, which has lasted for over two years. Natural compounds such as phenolic acids are being tested against Mpro and AAK1, which are key players in the SARS-CoV-2 life cycle. This research work aims to study the ability of a panel of natural phenolic acids to inhibit the virus's multiplication directly through Mpro and indirectly by affecting the adaptor-associated protein kinase-1 (AAK1). Pharmacophore mapping, molecular docking, and dynamic studies were conducted over 50 ns and 100 ns on a panel of 39 natural phenolic acids. Rosmarinic acid (**16**) on the Mpro receptor (− 16.33 kcal/mol) and tannic acid (**17**) on the AAK1 receptor (− 17.15 kcal/mol) exhibited the best docking energy against both receptors. These favourable docking score values were found to be superior to those of the co-crystallized ligands. Preclinical and clinical research is required before using them simultaneously to halt the COVID-19 life cycle in a synergistic manner.

## Introduction

**Figure 1:**
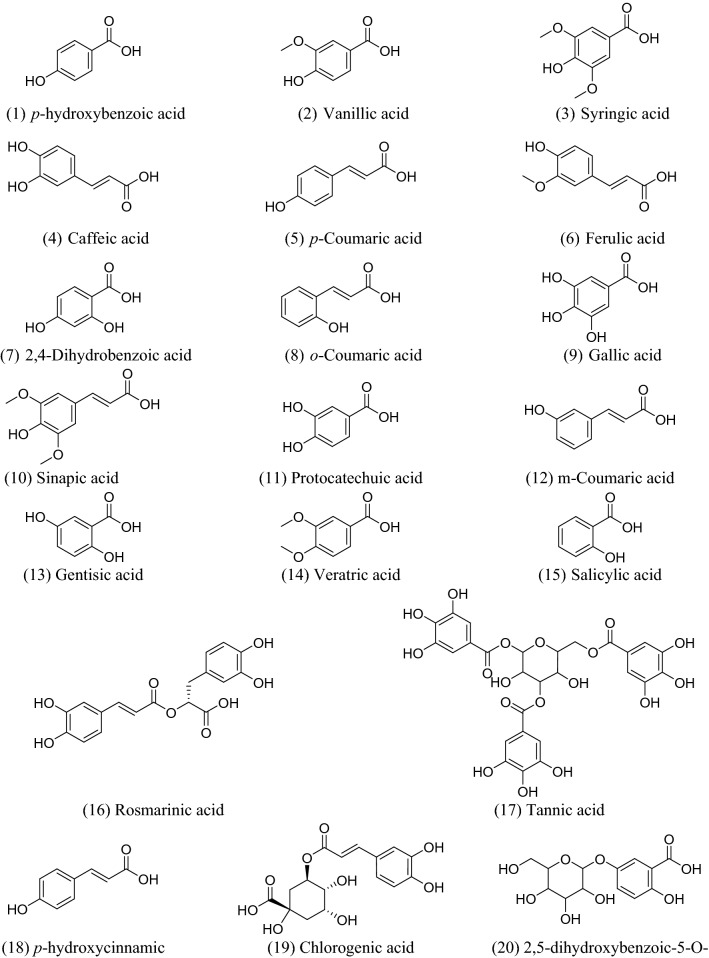

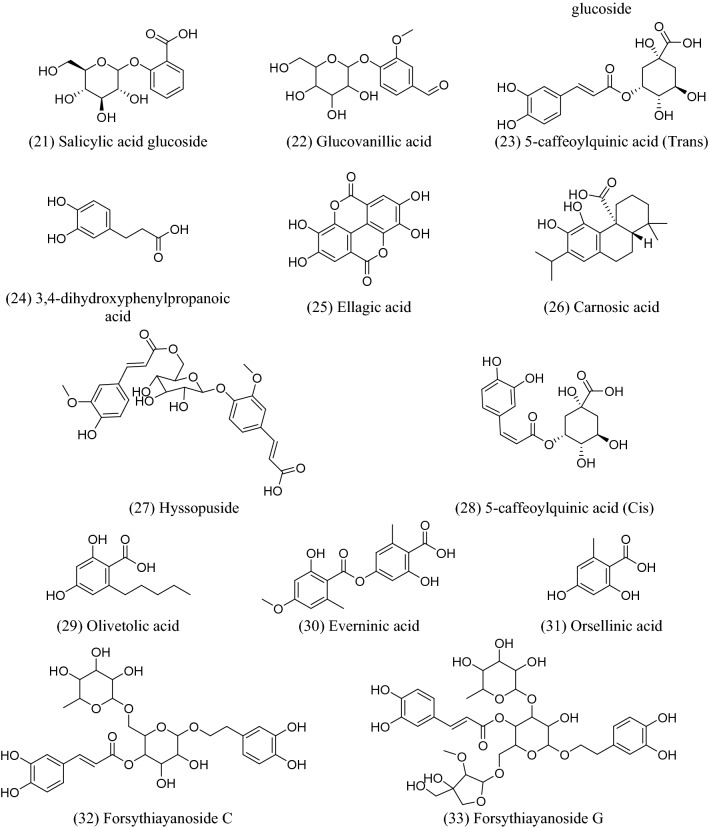

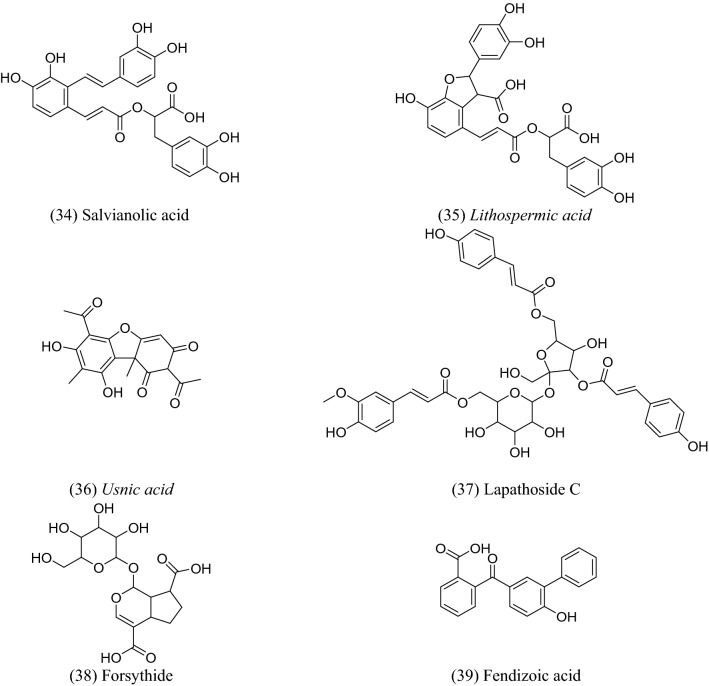
Natural phenolic acids that are investigated in this research work.

There are numerous potential sources of phenolic acids, such as fruits, vegetables, spices, and herbs^1^. These substances are naturally occurring by-products or secondary metabolites of plants, and they are found in food on a regular basis. They provide essential health benefits for people, including antioxidant, anti-inflammatory, anticancer, anti-allergic, antihypertensive, and antiviral capabilities^2^. Many studies have demonstrated the effectiveness of these substances in preventing viruses from causing serious health issues^3–5^. Natural phenolic acids and their derivatives have shown strong inhibitory effects on a variety of human viruses, including coronavirus (SARS-CoV-2)^6–10^. Based on the target, there are two groups of potential anti-coronavirus medications: one targets the human immune system or human cells, while the other focuses on the coronavirus itself^11,12^. SARS-CoV-2 has seven major structural proteins, and several human targets, such as transmembrane serine protease 2 (TMPRSS2), cathepsin L, and adaptor-associated protein kinase 1 (AAK1), are involved in the progression of symptoms linked to SARS-CoV-2 infections^14–16^. Phenolic acid products have been identified as excellent candidates as anti-SARS-CoV treatments, especially towards Mpro and AAK1^19,20^. Inhibition of AAK1 could be a good strategy to prevent SARS-CoV-2 entry. The carbonyl and hydroxyl groups of phenolic acids play a major role in forming good binding interactions with both Mpro and AAK1^21–23^. These findings have encouraged researchers to investigate natural phenolic acids against these two plausible targets in the SARS-CoV-2 life cycle, in an effort to find new natural and safe treatments for COVID-19. The selected library of natural phenolic acids is represented in Fig. 1.

## Methods

In this study, molecular operating environment (MOE, 2019.0102) software was used for pharmacophoric generation, searching and molecular docking^24–26^, energy minimized structures were gained by applying MMFF94x force field until RMSD gradient of 0.1 kcal mol^−1^ Å^−1^ was reached. The protein data bank (PDB) was the source of the X-ray crystallographic structures of SARS CoV-2 main protease enzyme (Mpro) (ID: 7AEH) and adaptor-associated protein kinase 1 (AAK1) (ID: 4WSQ). Triangle matcher as a placement method and London dG as a scoring algorithm were used to determine the pharmacophoric properties of both enzymes and the binding site in each protein file using the co-crystallized ligand. AutoDock Vina 1.1.2. Software was also used for conducting the molecular docking study to confirm the results. SwissADME was used to evaluate the ADME properties of the promising compounds^27^.

### Pharmacophore mapping

#### SARS CoV-2 main protease enzyme (Mpro)

The binding characteristics for the co-crystallized ligand were chosen, stored, and utilized to search for potential matches from the phenolic acids database using the PDB file (ID: 7AEH) that was obtained from the protein data bank.

#### Adaptor-associated protein kinase 1 (AAK1)

The PDB file (ID: 4WSQ) was obtained for mapping the pharmacophoric characteristics of AAK1 and examined to determine the most crucial binding properties. For the purpose of choosing the most appropriate phenolic acid structures, the created pharmacophore was preserved and utilized.

### Molecular docking

#### SARS CoV-2 main protease enzyme (Mpro)

The PDB file (ID: 7AEH) was used to identify the required key binding features for the ligand through analysis of the binding interactions of the co-crystallized ligand and to perform the docking procedure.

#### Adaptor-associated protein kinase 1 (AAK1)

PDB file (ID: 4WSQ) was used for performing molecular docking simulations, the tested compounds were docked at the binding pocket of the enzyme.

### Molecular dynamics simulations

The best docking postures were subjected to molecular dynamics (MD) computations to get insights into the stability of the protein–ligand interaction. The CHARMM-GUI solution builder created the input files for the MD calculations using the CHARMM force field parameters for proteins^28^. The simulation box dimensions of Mpro/Mpro-16 and AAK1/AAK1-16 were 98 × 98 × 98 nm and 132 × 132 × 132 nm, respectively, containing four sodium ions and seven chloride ions. The protein–ligand combination was subjected to the CHARMM36 forcefield. Prior to production simulation, the system's energy consumption was minimized using the steepest descent algorithm (5000 steps). The complex was then subjected to NVT and NPT ensemble, simulating for 125 ps at 300.15 K temperature utilizing 400 kJ mol^−1^ nm^2^ and 40 kJ mol^−1^ nm^2^ positional restrictions on the backbone and side chains, respectively, to equilibrate the complex for stabilizing its temperature and pressure. The complex is then put through a 100-ns production simulation run in an NPT ensemble at 300.15 K and 1 bar^29^.

### ADMET study

SwissADME (http://www.swissadme.ch/index.php) web server was used the study the ADME properties of the promising compounds, it can evaluate the pharmacokinetics, drug-likeness and medicinal chemistry friendliness of small molecules. The smiles of the selected library were generated and then the ADME properties were calculated. Lazar toxicity predictor web server (https://lazar.in-silico.ch/predict) was used to estimate the toxicity profile of (**16**) rosmarinic acid against Mpro and (**17**) tannic acid against AAK1.

## Results and discussions

### Pharmacophore mapping and molecular docking on SARS CoV-2 main protease (Mpro)

#### Pharmacophore generation

**Figure 2:**
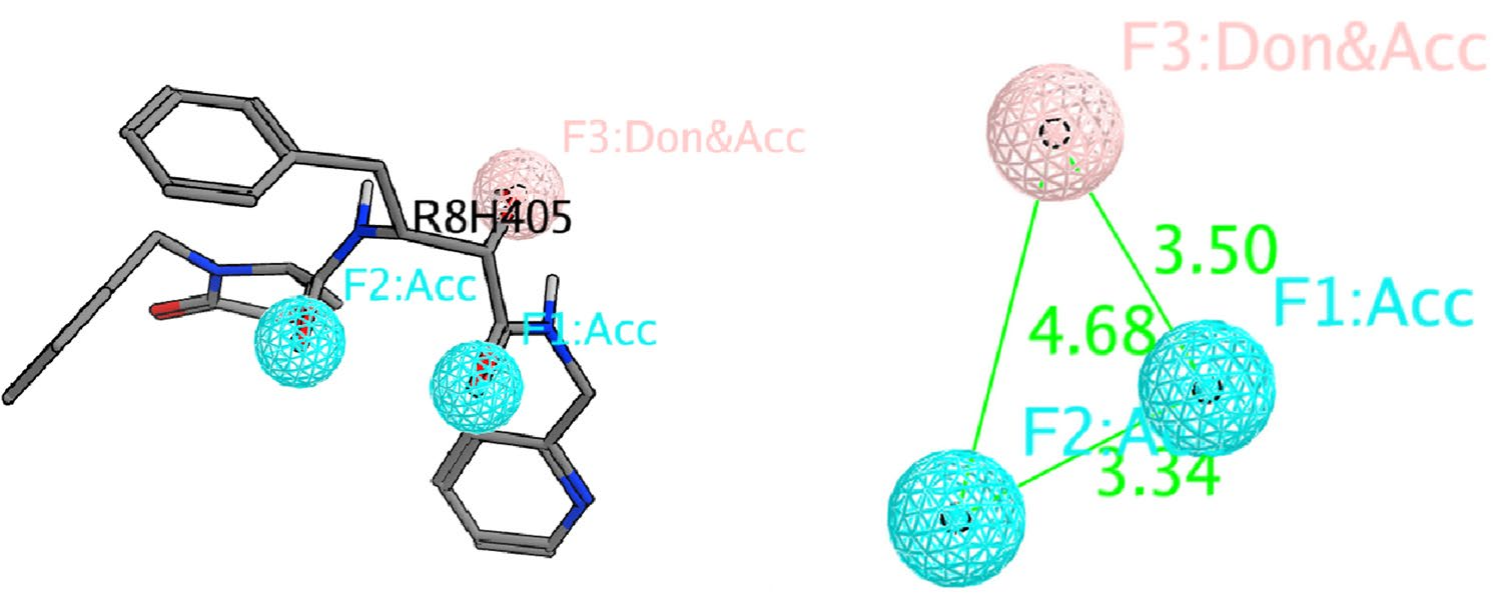
Pharmacophoric features for SARS CoV-2 Mpro obtained from the co-crystallized ligand (PDB: 7AEH) (Acc: Acceptor/Don: Donor).

3D Pharmacophores represent the ensembles of the chemically defined interactions of the bioactive conformation of the ligand, accordingly, pharmacophore generation represents an elegant way to decode the chemically encoded ligand information and so becomes a valuable tool in drug design^30,31^. The selected PDB file for Mpro enzyme with its co-crystallized ligand; R8H, (2 ~ {R})-5-oxidanylidene- ~ {N}-[(2 ~ {R},3 ~ {S})-3-oxidanyl-4-oxidanylidene-1-phenyl-4-(pyridin-2-ylmethylamino)butan-2-yl]-1-(phenylmethyl)pyrrolidine-2-carboxamide [C28 H30 N4 O4] was used to identify the essential pharmacophoric features for better binding with the enzyme. The key amino acids in Mpro active site were HIS-41 and CYS-145 in the catalytic dyad^32,33^ in addition to GLY-143 that interacts with the oxygen atoms of the co-crystallized ligand. The pharmacophoric features were selected as shown in Fig. 2.

**Figure 3:**
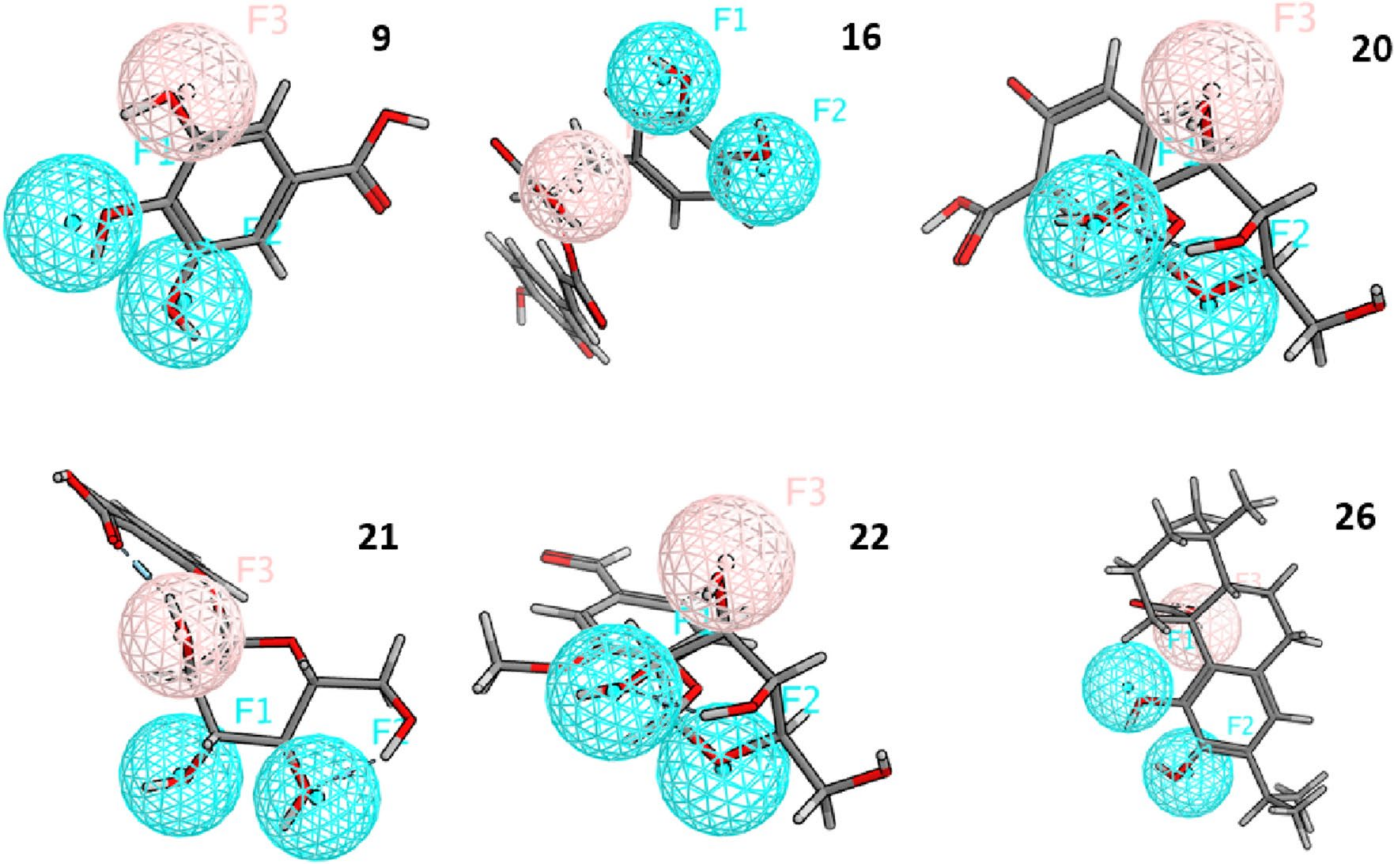
Fitting of the best six hits on the generated Mpro pharmacophore.

Virtual screening of the selected database against these pharmacophoric features revealed that six hit molecules, (**9**) gallic acid, (**16**) rosmarinic acid, (**20**) 2,5-dihydroxybenzoic-5-O-glucoside, (**21**) salicylic acid glucoside, (**22**) glucovanillic acid and (**26**) carnosic acid, they have showed RMSD value of 0.53, 0.56, 0.29, 0.18, 0.29 and 0.54 respectively, Fig. 3 is showing that these six hits are fitting the generated pharmacophoric features in Mpro.

#### Molecular docking on SARS Cov-2 Mpro

Molecular docking study was carried out for the hit molecules, their binding energy scores are summarized in Table 1, compared with the co-crystallized ligand (− 10.52 kcal./mol.).

**Table 1:** Binding energy scores for the tested acids (Mpro enzyme).

Compound no	Energy score using MOE (kcal/mol)	Energy score using Autodock Vina (kcal/mol)
**9**	− 9.78	− 9.20
**16**	− 16.33	− 14.53
**20**	− 12.28	− 12.15
**21**	− 9.81	− 9.65
**22**	− 9.53	− 8.43
**26**	− 11.08	− 11.02
Co-Cry. ligand	− 10.52	− 10.92

**Figure 4:**
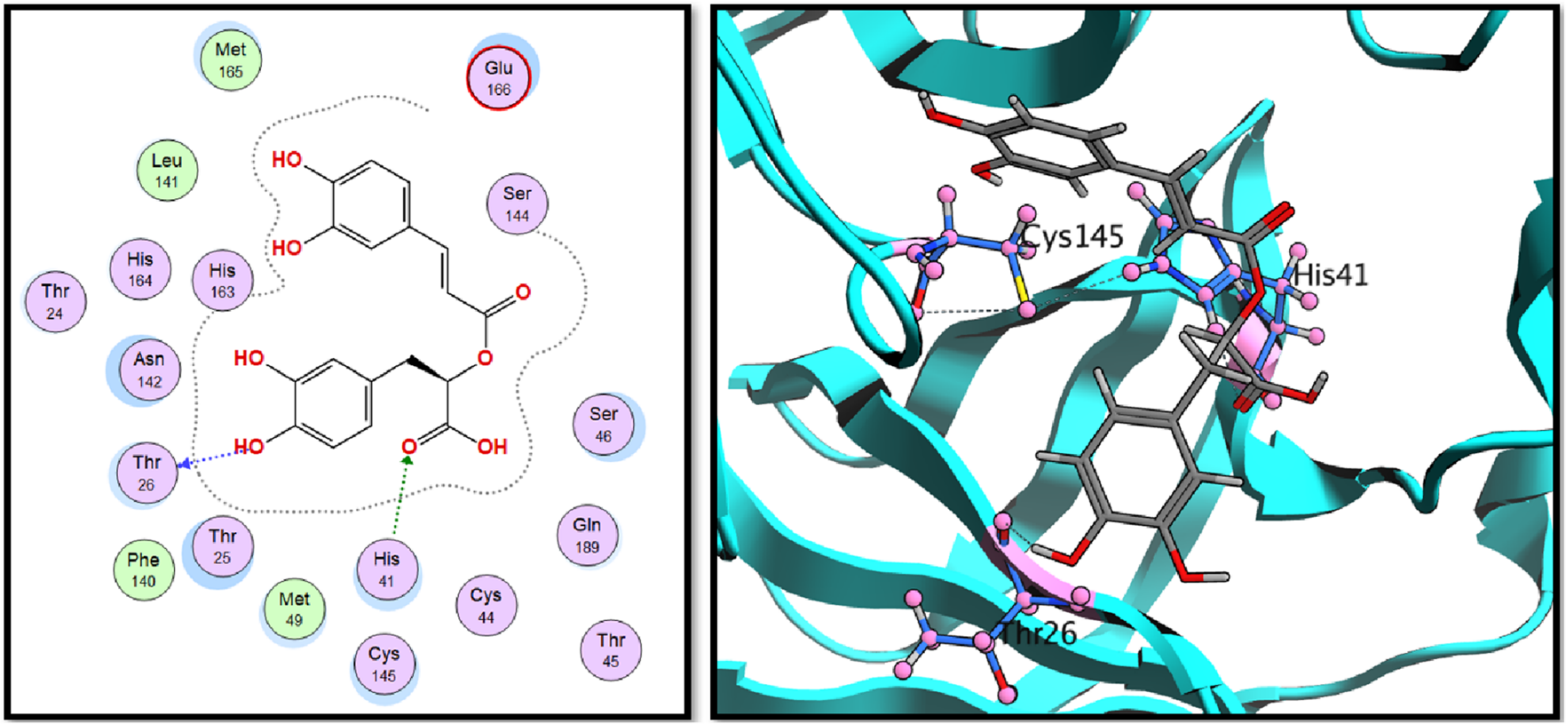
2D and 3D interactions of rosmarinic acid (**16**) in the binding pocket of Mpro enzyme.

**Figure 5:**
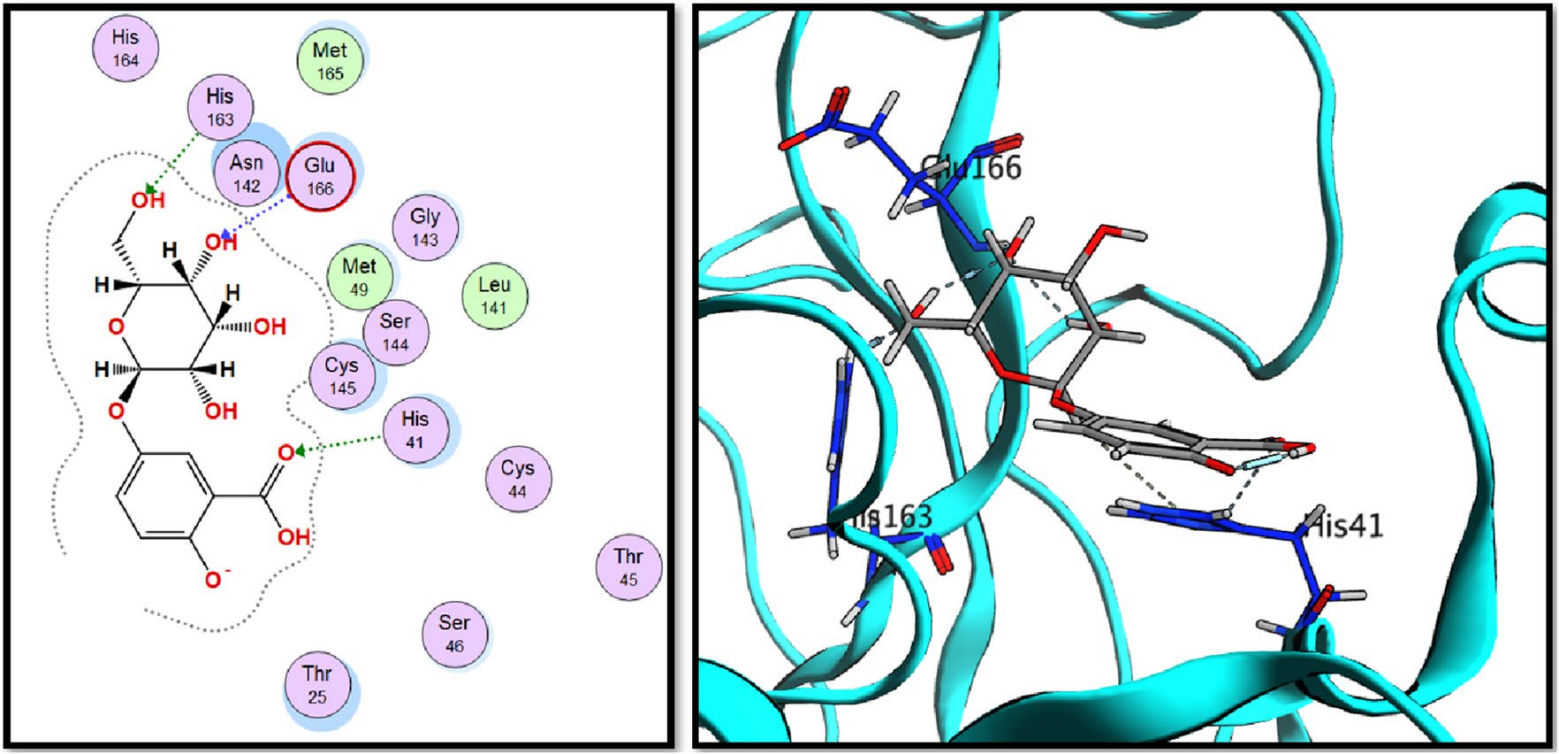
2D and 3D interactions of 2,5-dihydroxybenzoic-5-O-glucoside (**20**) in the binding pocket of Mpro enzyme.

**Figure 6:**
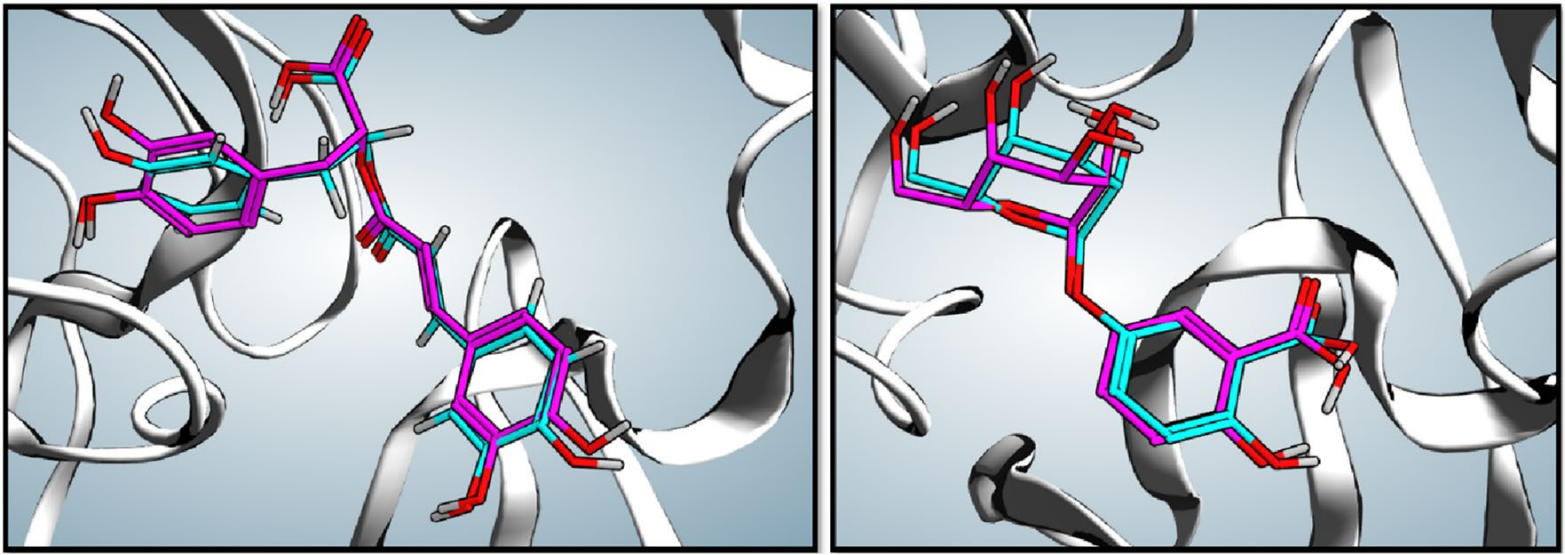
Alignment of rosmarinic acid (**16**) and 2,5-dihydroxybenzoic-5-O-glucoside (**20**) docking poses obtained from MOE (cyan) with that obtained from Autodock Vina (purple).

According to the binding energy scores in Table 1, both compounds **16** (rosmarinic acid) and **20** (2,3-dihydroxybenzoic-5-O-glucoside) showed the highest scores, − 16.33 and − 12.28 kcal./mol. Both binding interactions are well demonstrated in Figs. 4 and 5, rosmarinic acid interacts with the catalytic dyad through HIS-41 with its carboxylic acid carbonyl oxygen, while compound **20** (2,5-dihydroxybenzoic-5-O-glucoside) showed the same binding interaction revealing its expected good inhibitory activity on Mpro enzyme. To validate the docking results, the best pose of the tested compounds (**16**) and (**20**) using MOE was aligned with the poses obtained from AutoDock Vina (Fig. 6).

### Pharmacophore mapping and molecular docking on adaptor-associated protein kinase 1 (AAK1)

#### Pharmacophore generation

**Figure 7:**
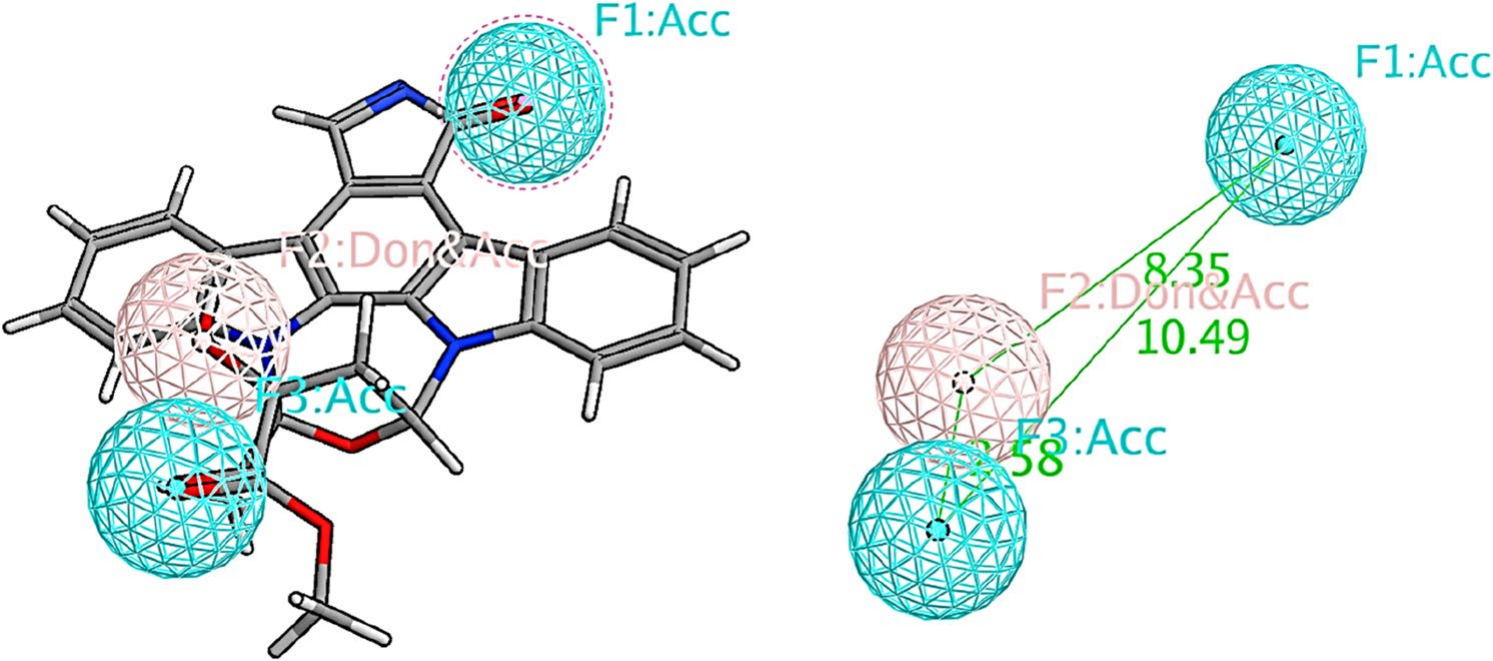
Pharmacophoric features for AAK1 enzyme obtained from the co-crystallized ligand (PDB: 4WSQ).

**4WSQ** PDB file was used to identify the key pharmacophoric features of AAK1 enzyme with its co-crystallized ligand (KSA, K-252A [C_27_H_21_N_3_O_5_]). The pharmacophoric features were selected (Fig. 7).

**Figure 8:**
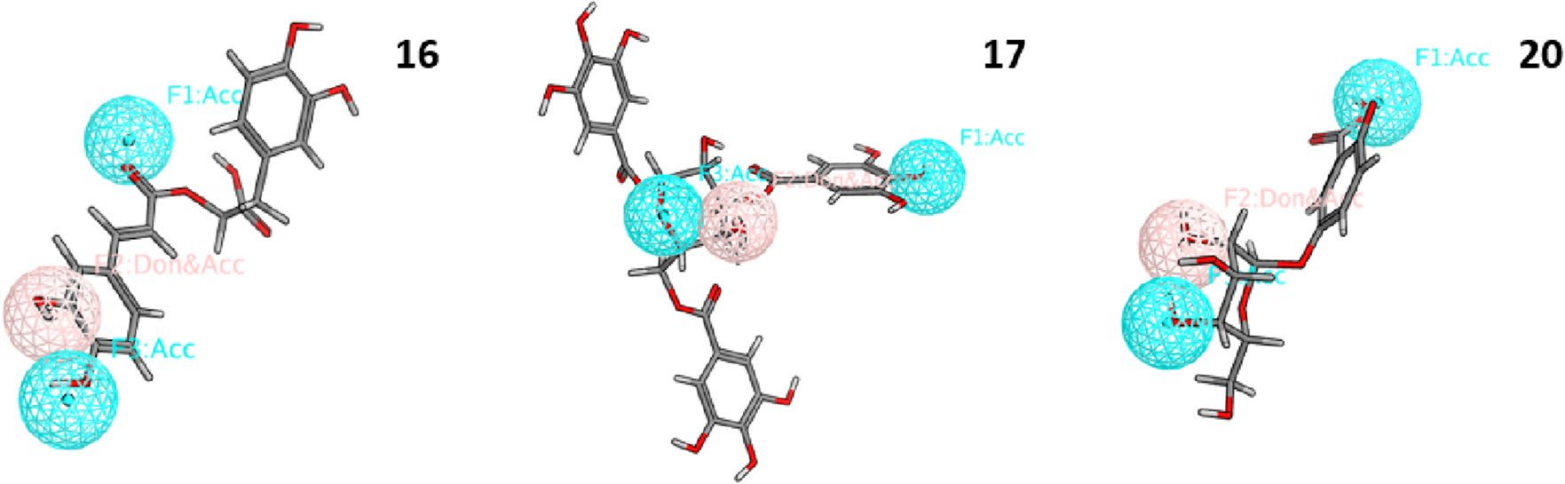
Fitting of the three hit compounds to the generated AAK1 pharmacophoric features.

Virtual screening was performed on the generated pharmacophore model, it has showed three hit molecules: (**16**) rosmarinic acid, (**17**) tannic acid and (**20**) 2,3-dihydroxybenzoic-5-O-glucoside with RMSD values of 0.45, 0.20, 0.28, respectively (Fig. 8).

#### Molecular docking against AAK1

Molecular docking study was carried out for the hit molecules, their binding energy scores are summarized in Table 2, compared to the co-crystallized ligand (− 13.28 kcal./mol.).

**Table 2:** Binding energy scores for the tested acids (AAK1 enzyme).

Compound no.	Energy score (kcal/mol)	Energy score using Autodock Vina (kcal/mol)
**16**	− 10.68	− 10.52
**17**	− 17.15	− 16.21
**20**	− 9.40	− 9.33
Co-crystallized ligand	− 13.28	− 13.15

**Figure 9:**
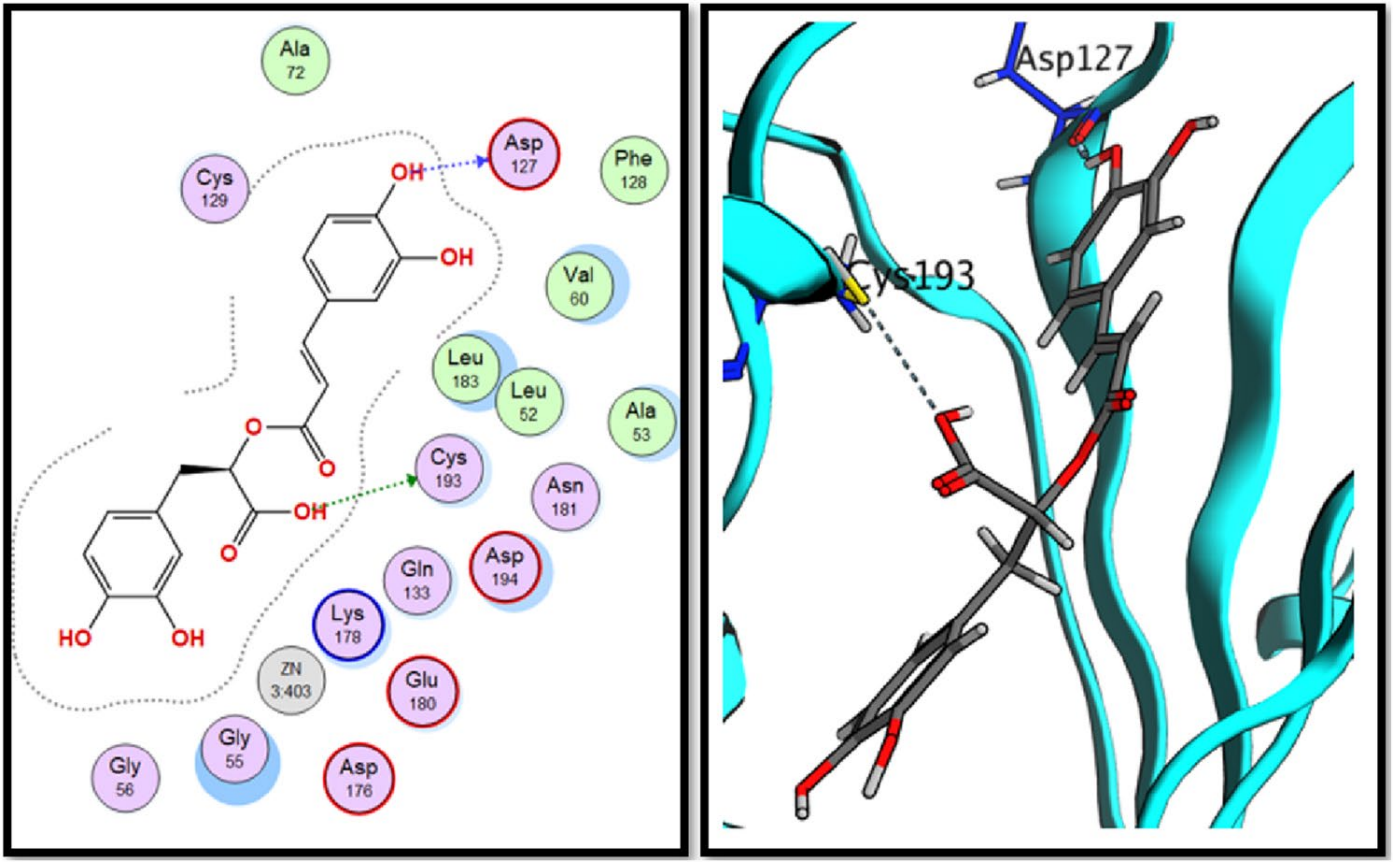
2D and 3D interactions of rosmarinic acid (**16**) in the AAK1 active site.

**Figure 10:**
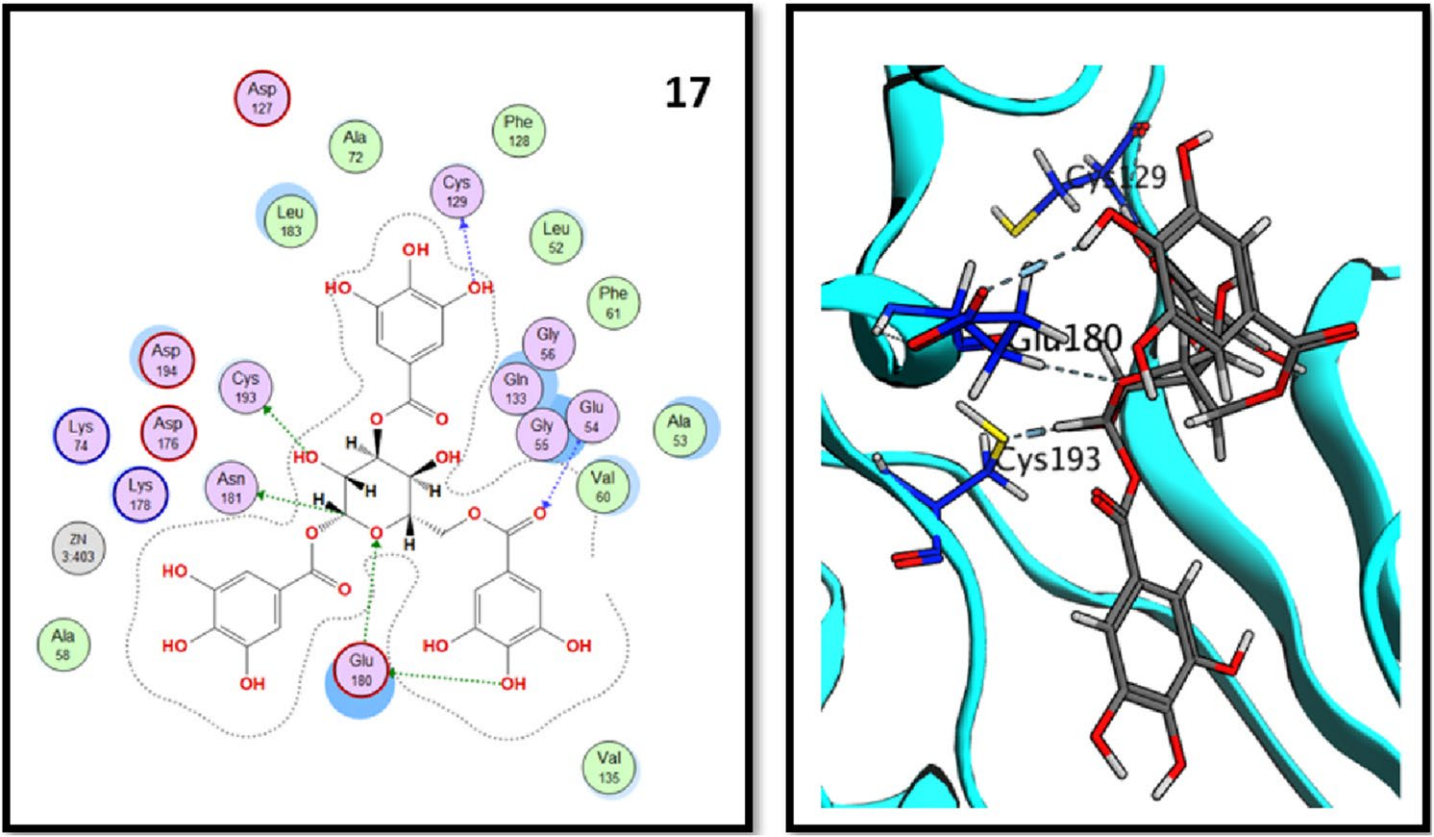
2D and 3D interactions of tannic acid (**17**) in the AAK1 active site.

**Figure 11:**
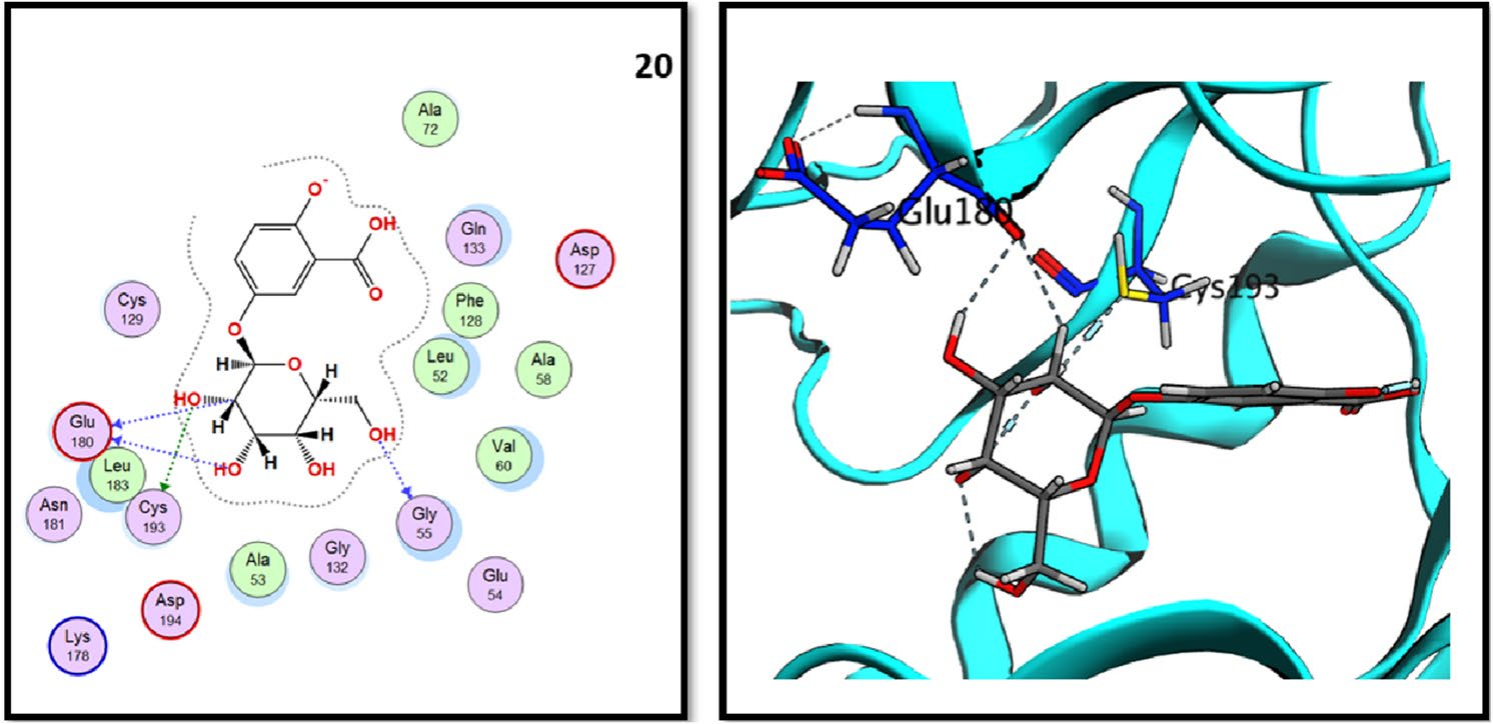
2D and 3D interactions of 2,5-dihydroxybenzoic-5-O-glucoside (**20**) in the AAK1 active site.

Analysis of molecular docking results revealed that all three tested compounds have the main binding interaction with CYS-193^34^. Compound (**17**), tannic acid showed the highest binding energy score (− 17.15 kcal./mol.) with strong H-bond interactions through its hydroxyl groups to CYS-129, GLU-180 and CYS-193 amino acid residues. On the other hand, compound (**16**), rosmarinic acid showed H-bond interaction through its carboxylic acid OH group to CYS-193 in addition to binding of phenolic OH group to ASP-127 in the binding vicinity. Finally, compound (**20**), 2,3-dihydroxybenzoic-5-O-glucoside showed the H-bond interactions with GLU-180 and CYS-193 residues through its hydroxyl groups. All obtained results are illustrated in Figs. 9, 10 and 11.

### Molecular dynamics (MD) simulation

The GROMACS software was used to run an MD simulation in order to compare the behaviour of compound (**16**) rosmarinic acid with MPRO and compound (**17**) tannic acid with AAK1 across simulation times of 50 and 100 ns, respectively^35–37^.

#### Simulation time over 50 ns

##### Analysis of the root mean square deviation (RMSD)

**Figure 12:**
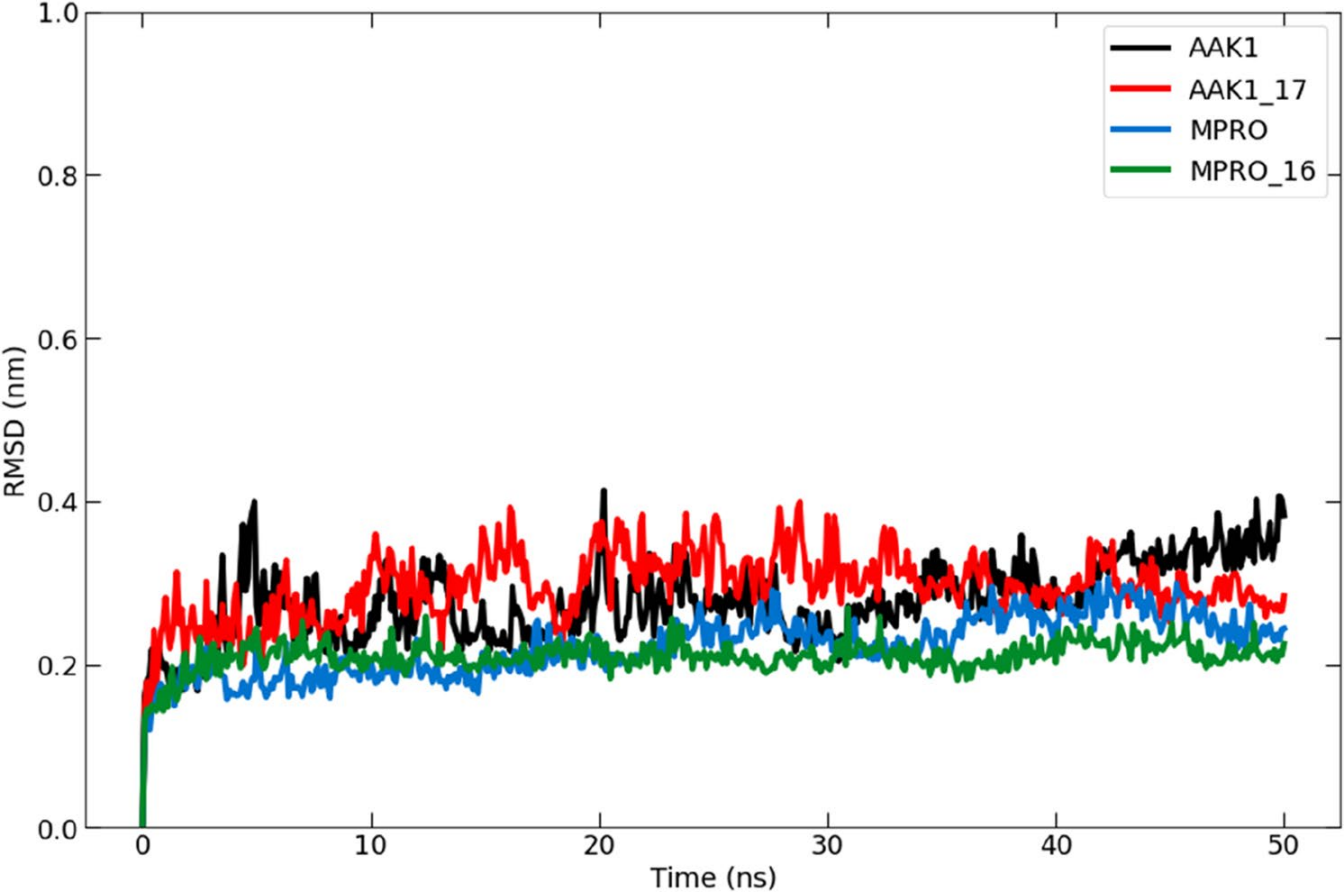
A depiction of the root mean square deviation (RMSD) from the molecular dynamics simulation trajectories of the Mpro (blue) and Mpro-**16** complex (green) in addition to AAK1 (black) and AAk1-**17** complex (red) during a 50-ns MD simulation.

The root mean square deviation (RMSD) was investigated quantitatively to assess the degree of divergence of each complex protein structure with each ligand from its baseline behaviour. The system's stability is evaluated during the simulation with the help of the RMSD. In two different MD simulations, a control system (a ligand-free structure) and a complex were put up for this^38^. The stability and convergence of compound **16** in the target Mpro and compound **17** in the target AAK1 were investigated using a 50-ns molecular dynamics (MD) simulation, where the backbone atoms' RMSD value was obtained, as shown in Fig. 12. The findings indicated that the complex-maintained equilibrium for the course of the experiment. The RMSD values of the apoprotein and the compound **17**-bound complex were 0.14–0.36 nm. Furthermore, the RMSD values of the apoprotein and the compound **16**-bound complex ranged from 0.12 to 0.23 nm. Compounds **16** and **17** behaved consistently inside their pockets throughout the simulation and went farther toward the binding pocket. This could explain why both **16** and **17** have potent inhibitory effects against Mpro and AAK1, respectively.

##### Analysis of the root mean square fluctuation (RMSF)

**Figure 13:**
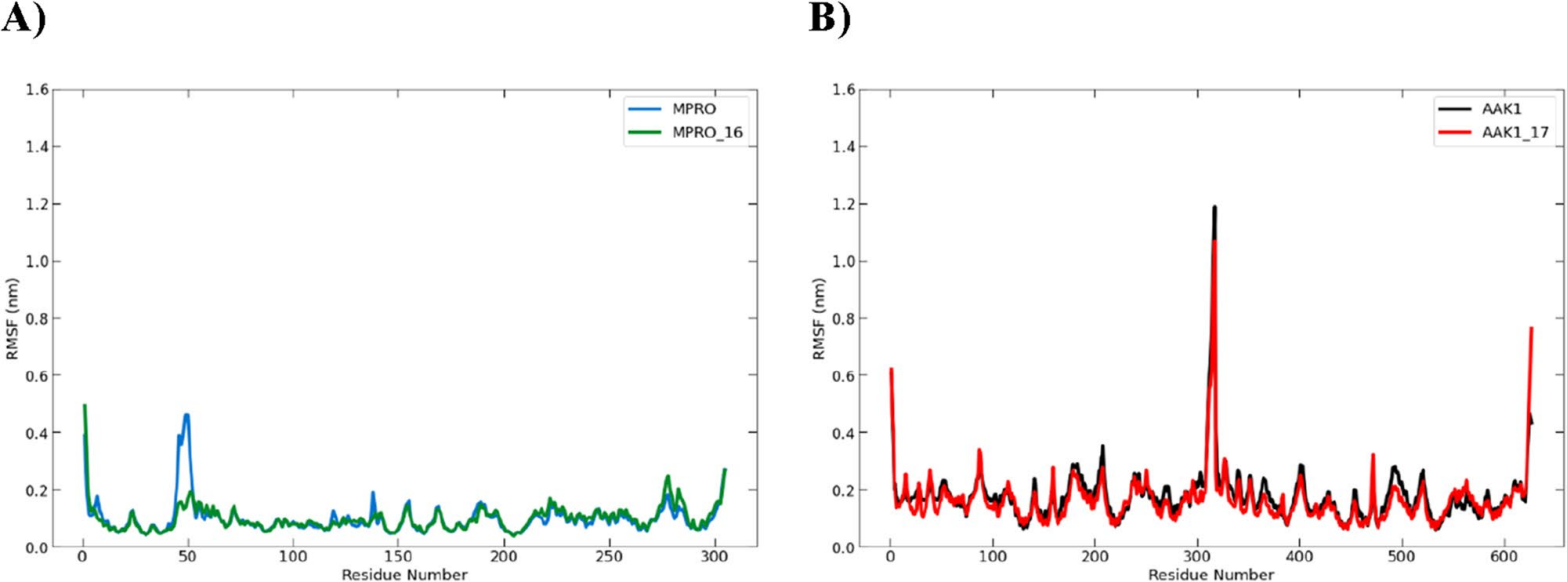
Plot of the root mean square fluctuation (RMSF) for (**A**) 300-ns molecular dynamics simulation of the Mpro protein (blue) and Mpro-**16** complex (green) and (**B**) 600-ns molecular dynamics simulation of the AAK1 protein (black) and AAK1-**17** complex (red).

The local changes in the protein structure brought on by the presence of the suggested inhibitor were examined using the root mean square fluctuation (RMSF)^39^. Throughout the simulation period, it showed how flexible the protein was. The ranges of 0.03–0.48 nm and 0.15–1.2 nm showed the highest variation. The similar residues in the compounds **16** or **17**-bound complexes were less flexible than the native, unbound targets in general. These low-fluctuating residues contributed to the stability of the docked molecules at the binding site (Fig. 13).

##### Analysis for the radius of gyration (R_g_)

**Figure 14:**
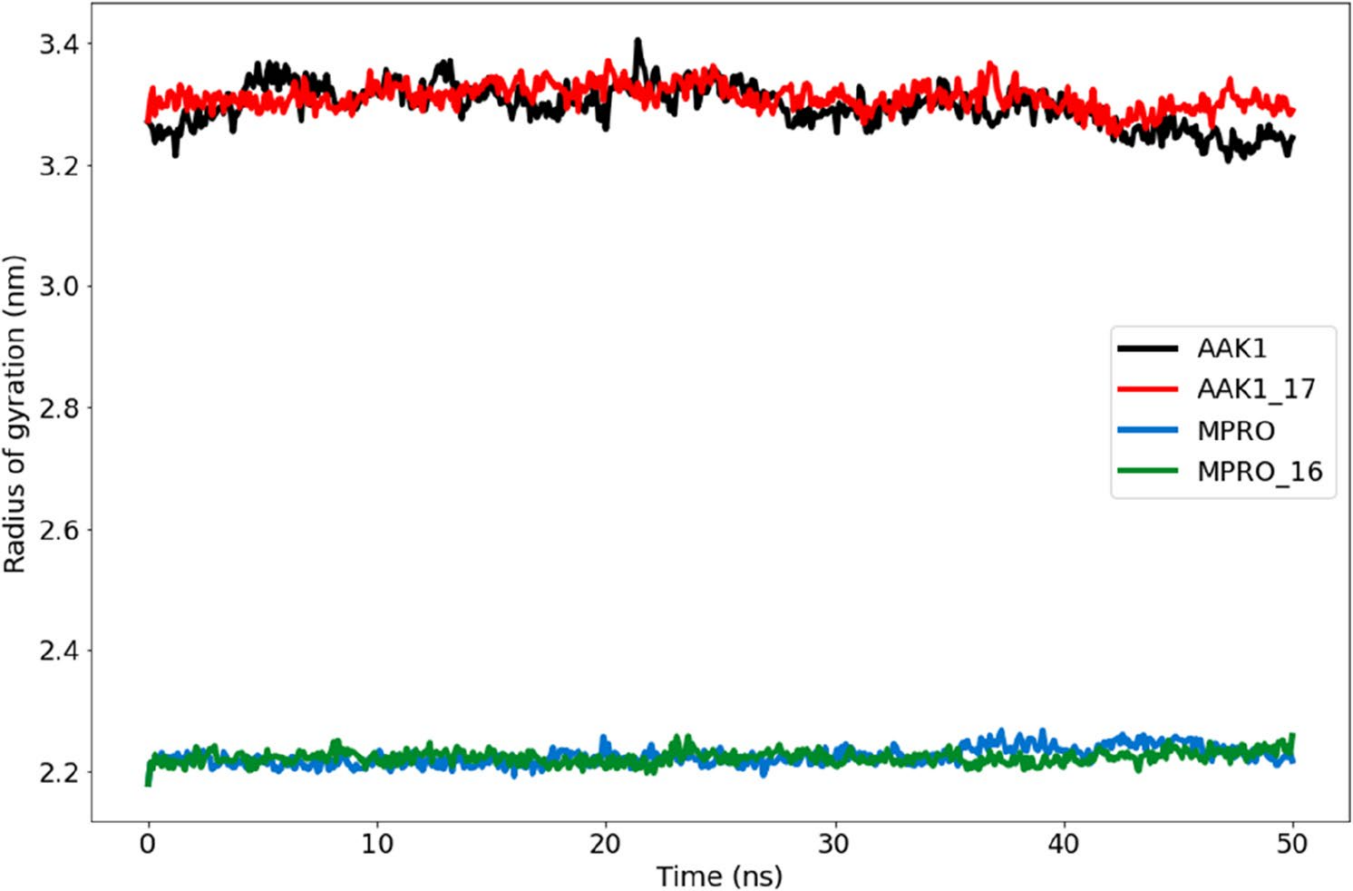
A plot illustrated the radius of gyration (R_g_) for the Mpro protein (blue) and compound **16** complex (green) in addition to the AAK1 protein (black) and compound **17** complex (red) during the 50-ns molecular dynamics simulation.

The radius of gyration provides information regarding the size and compactness of protein molecules (Rg). The Rg can be used to track the folding and unfolding of protein structures when ligands are bound^40^. Rg values for the drug-bound complexes were typically closer to the native unbound Mpro and AAK1 values (Fig. 14). Compound 16 and Mpro had average Rg values of 2.18–2.26 nm, whereas compound **17** and AAK1 had values of 3.22–3.40 nm. A less compact or more unfolded protein–ligand interaction is indicated by a greater Rg. A protein is considered securely folded, though, if its Rg value holds steady during the MD simulation. It is viewed as unfolded if the value of Rg changes with time. Figure 14 illustrates how, as compared to the unbound protein, each complex had very identical properties in terms of compactness and almost constant values of Rg.

##### Analysis of solvent-accessible surface area (SASA)

**Figure 15:**
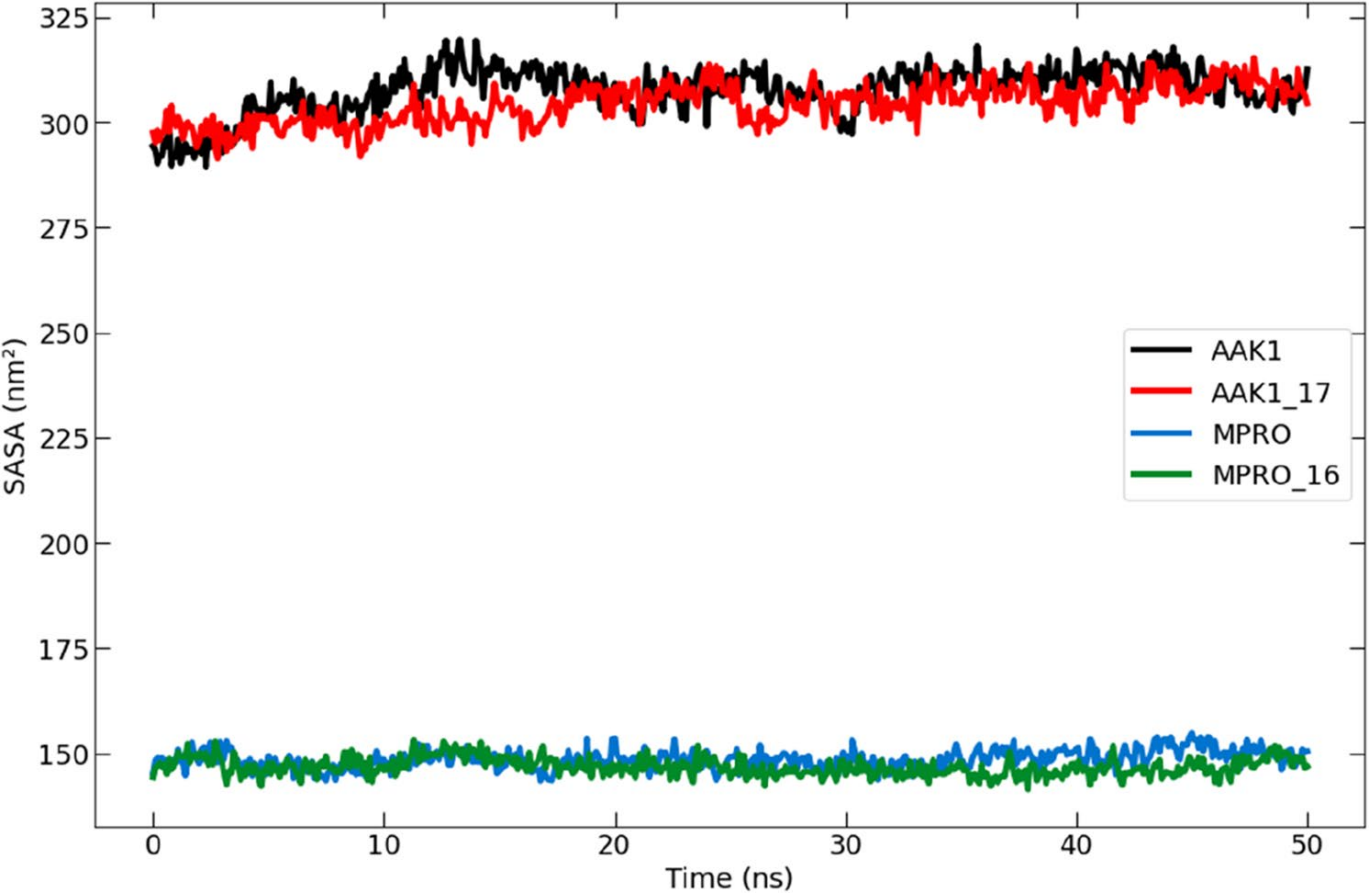
A graph showing the solvent-accessible surface area (SASA) for the Mpro protein (blue) and the complex of compound **16** (green) in addition to the AAK1 protein (black) and the complex of compound **17** (red) during the 50-ns molecular dynamics simulation.

Both with and without ligands, the protein's solvent-accessible surface area (SASA) was studied. The protein–ligand complex's SASA computation is used to forecast the number of conformational changes that the aqueous solvent can access^41^. As a result, the SASA was used to evaluate interactions between the complex and the solvent throughout the 50-ns MD simulation. For the unbound protein and protein–ligand complexes, Fig. 15 shows the SASA vs. simulation time curve. In addition to compound **17** and AAK1, the SASA averages for compound **16** and Mpro ranged from 141 to 155 nm^2^ and 290 to 320 nm^2^, respectively. Each compound binding caused the SASA to slightly increase due to the expanded surface generated by a portion of the bound ligand surface poking out from the protein surface.

##### Analysis of hydrogen bond

**Figure 16:**
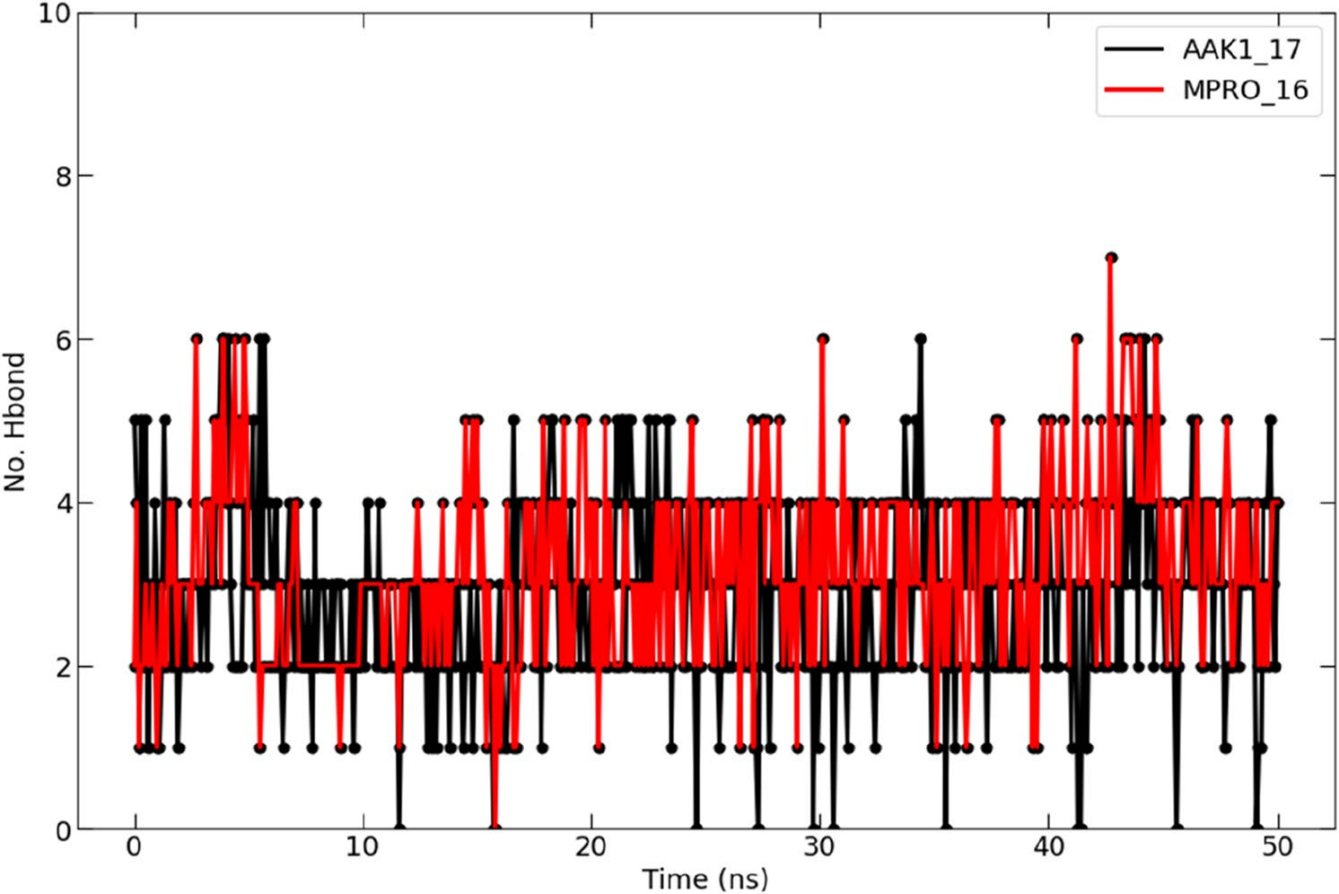
A graph presenting the numbers of hydrogen bonds that were seen in the complex formed by compound **16** (with Mpro in red color) and compound **17** (with AAK1 in black color) during the 50-ns molecular dynamics simulation.

The protein–ligand complex is stabilized by hydrogen bonds that form between the receptor and ligand. It also impacts the design of medications and their specificity, metabolization, and adsorption^42^. Therefore, the hydrogen bonds in each ligand–protein combination were studied. Figure 16 displays the total number of hydrogen bonds discovered in the complex after a 50-ns simulation. Each complex had between one and six hydrogen bonds, with two of them remaining constant over the course of the simulation. Additionally, each compound showed a similar hydrogen-bonding pattern throughout the simulation, as seen in Fig. 16.

#### Simulation time over 100 ns

##### Analysis of the root mean square deviation (RMSD)

**Figure 17:**
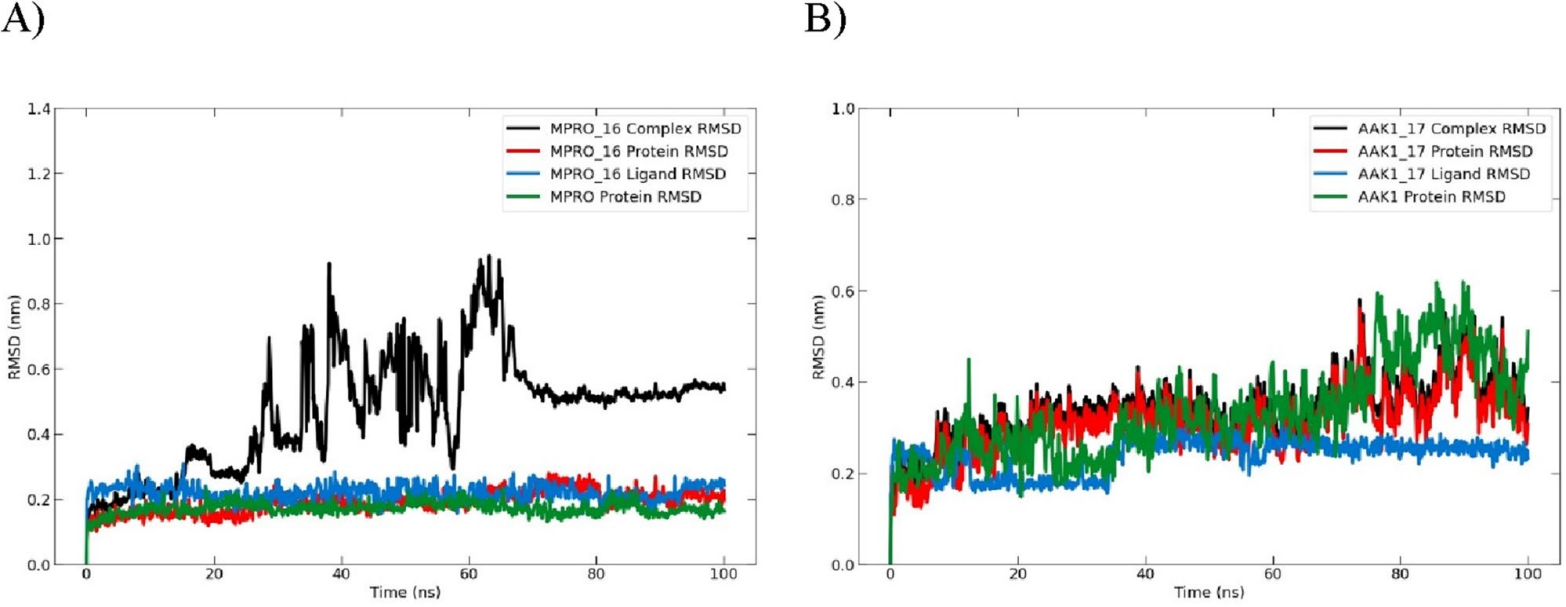
Plot of the root mean square deviation (RMSD) for (**A**) 100-ns molecular dynamics simulation of the Mpro protein and Mpro-**16** complex and (**B**) 100-ns molecular dynamics simulation of the AAK1 protein and AAK1-**17** complex.

When the simulation period was extended to 100 nm, we found that the complex still maintained equilibrium for the course of the experiment (Fig. 17). The RMSD values of the apoprotein and the compound **17**-bound complex reached 0.58 nm. Furthermore, the RMSD values of the apoprotein and the compound **16**-bound complex reached 0.29 nm. So, compounds **16** and **17** still behaved consistently inside their pockets throughout the simulation and went farther toward the binding pocket.

##### Analysis of the root mean square fluctuation (RMSF)

**Figure 18:**
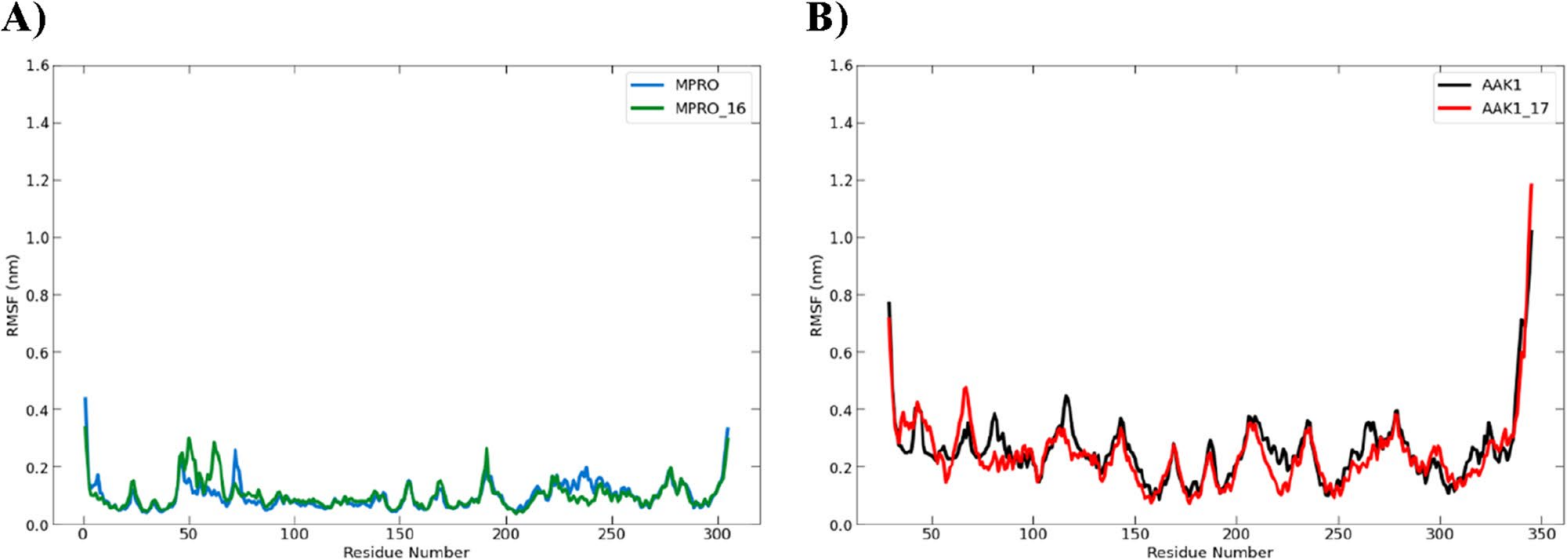
Plot of the root mean square fluctuation (RMSF) for (**A**) 300-ns molecular dynamics simulation of the Mpro protein (blue) and Mpro-**16** complex (green) and (**B**) 350-ns molecular dynamics simulation of the AAK1 protein (black) and AAK1-**17** complex (red).

Throughout the extended simulation period to 100 ns, it still showed how flexible the proteins were. The similar residues in the compounds **16-** and **17**-bound complexes were less flexible than the native, unbound targets. These low-fluctuating residues contributed to the stability of the docked molecules at the binding site (Fig. 18).

##### Analysis for the radius of gyration (R_g_)

**Figure 19:**
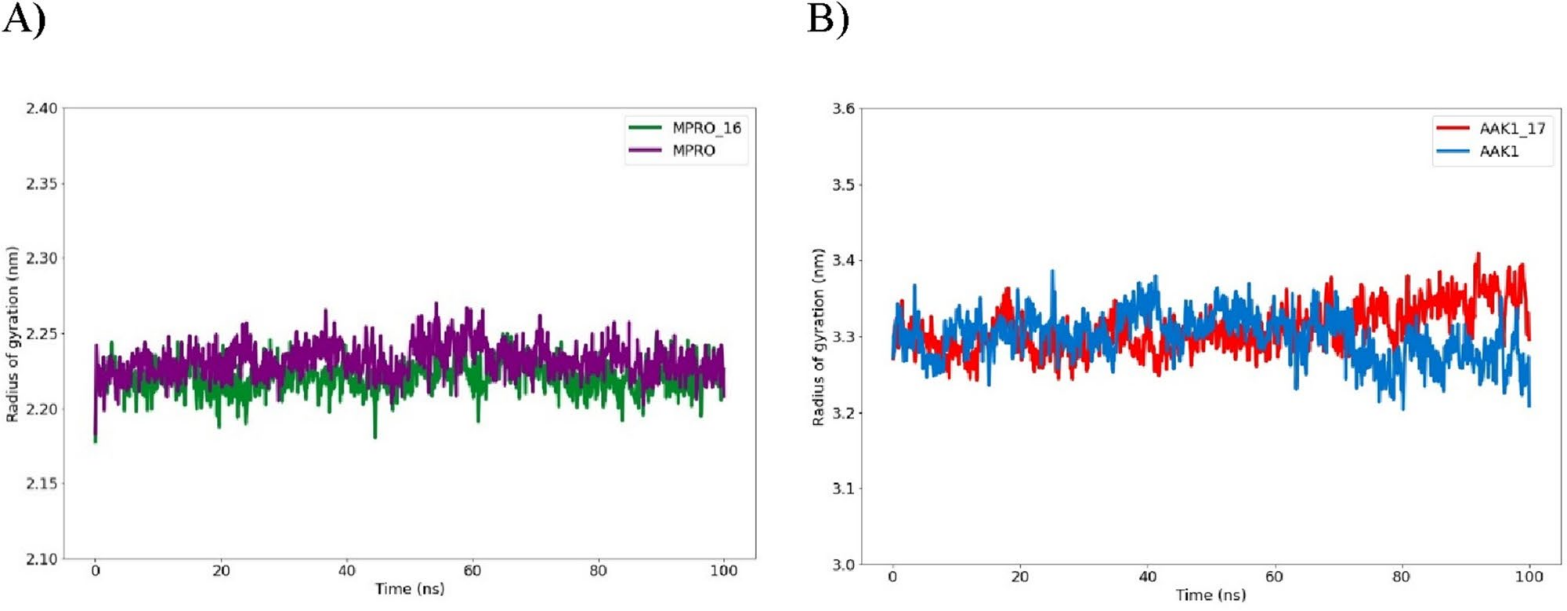
A plot illustrated the radius of gyration (R_g_) for (**A**) the Mpro protein and compound **16** complex in addition to the AAK1 protein and (**B**) compound **17** complex during the 100-ns molecular dynamics simulation.

Rg values for the drug-bound complexes were still typically closer to the native unbound Mpro and AAK1 values. Figure 19 illustrates how, as compared to the unbound protein, each complex had very identical properties in terms of compactness and almost constant values of Rg.

##### Analysis of solvent-accessible surface area (SASA)

**Figure 20:**
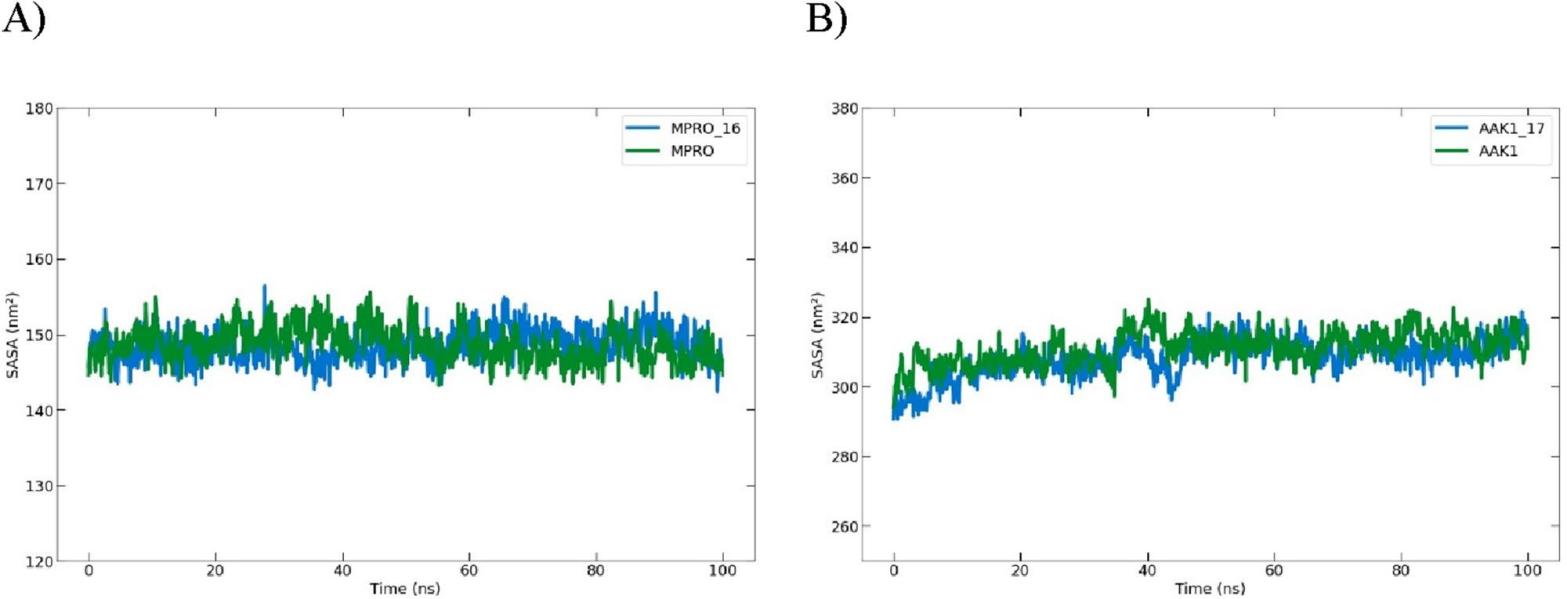
A graph showing the solvent-accessible surface area (SASA) for (**A**) the Mpro protein and the complex of compound **16**, and (**B**) the AAK1 protein and the complex of compound **17** during the 100-ns molecular dynamics simulation.

Each compound binding still caused SASA to increase slightly due to the expanded surface resulting from a portion of the binding surface bound from the protein surface (Fig. 20).

##### Analysis of hydrogen bond

**Figure 21:**
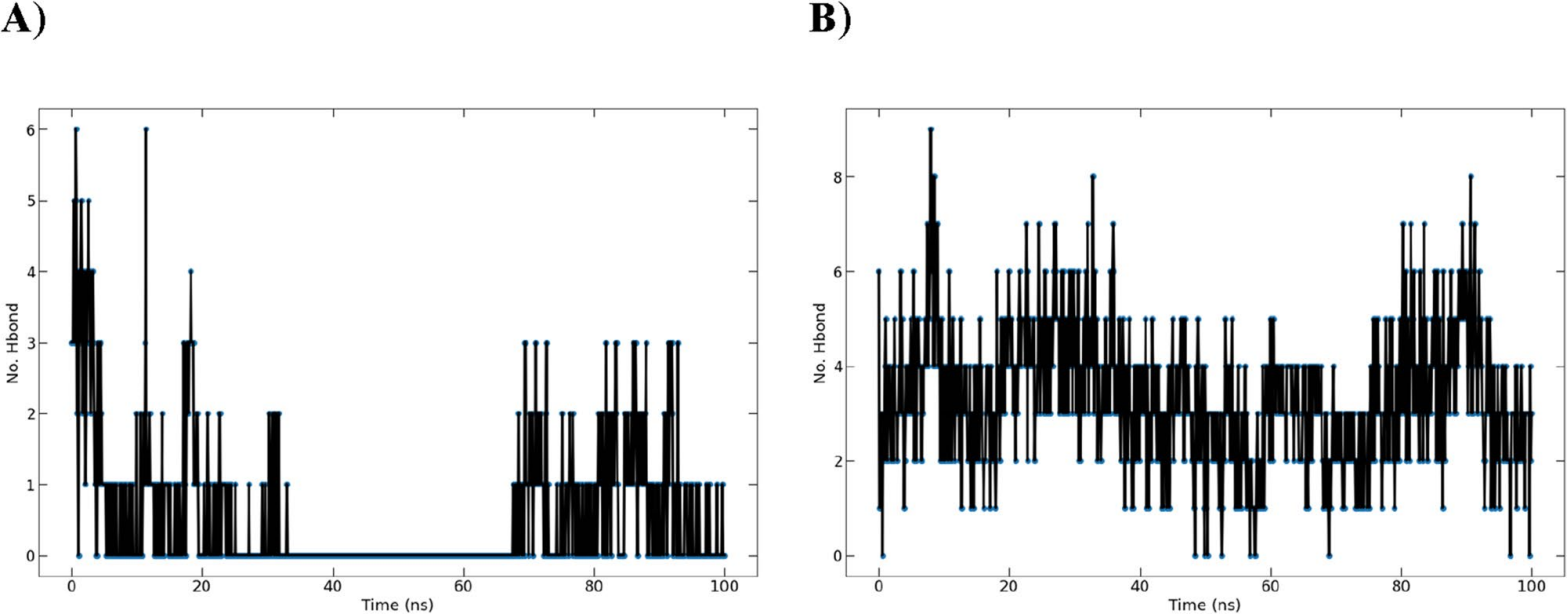
A graph presenting the numbers of hydrogen bonds that were seen in the complex formed by (**A**) compound **16** (with Mpro in red color) and (**B**) compound **17** (with AAK1 in black color) during the 100-ns molecular dynamics simulation.

The complex formed by compound **17** with AAK1 (in black color) still had between one and six hydrogen bonds, with two of them remaining constant over the course of the simulation (100 ns). But the complex formed by compound **16** with Mpro (in red color) had between one and three hydrogen bonds, without making any hydrogen bonding around 30–70 ns period **(**Fig. 21).

##### Principle component analysis (PCA)

**Figure 22:**
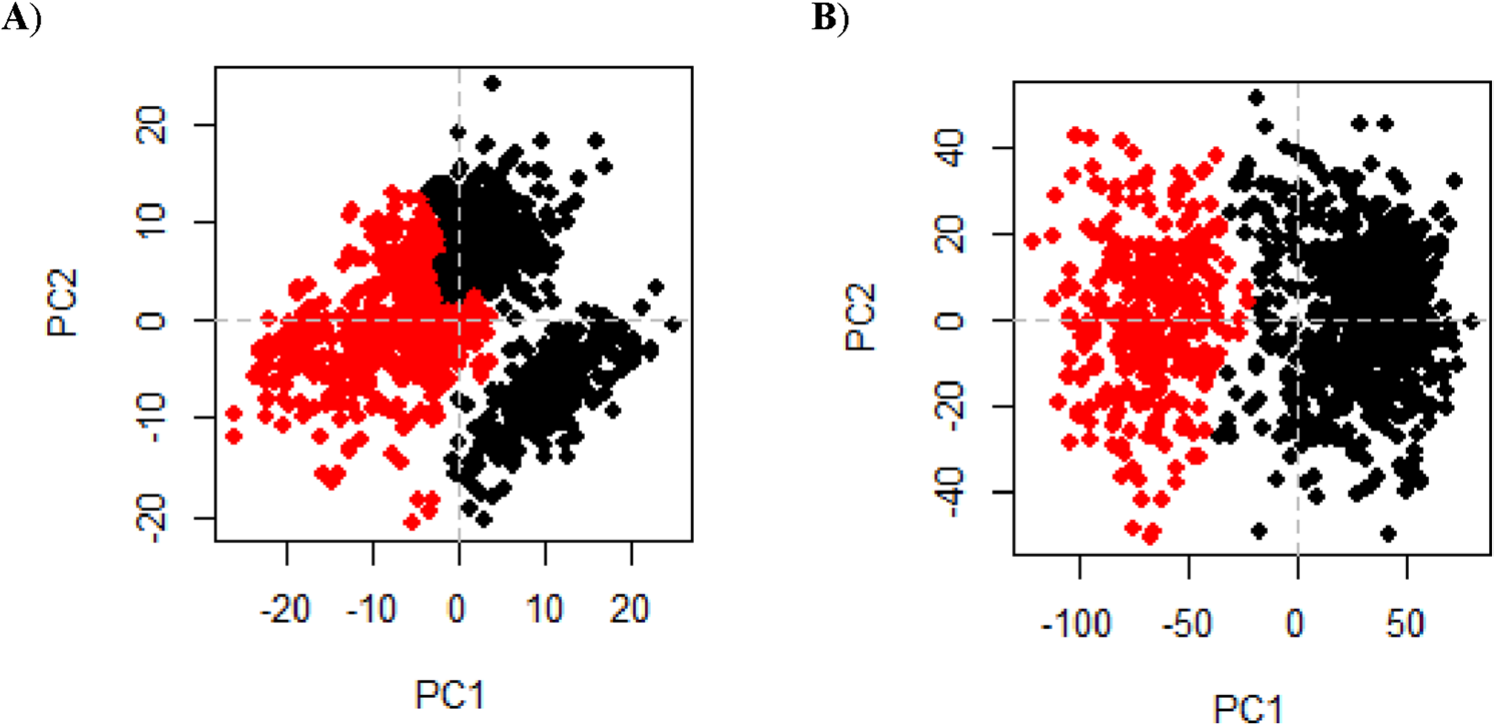
First two eigenvectors describe the protein motion in phase space for (**A**) Mpro-16 and (**B**) AAK1-17 complexes.

The conformational distribution during the simulation time was investigated using the PCA approach, as well as the large-scale collective motions of the protein in protein–ligand complexes on the simulation-generated trajectories. It was assumed that the complex that takes up less phase space with a stable cluster is more stable than the complex that takes up more space with a nonstable cluster^43^. During simulations of compound 16 bound to Mpro and compound 17 bound to AAK1 proteins, the first two principal components (PC1 and PC2) were chosen to investigate their projection of trajectories in phase space. The results clearly showed that Mpro-16 complex occupied smaller regions of phase space than AAK1-17 complex (Fig. 22).

##### Binding energy estimation by MM/PBSA method

The Molecular Mechanics/Poisson Boltzmann Surface Area (MM/PBSA) method throughout the 100 ns simulations was selected for rescoring complexes because it is the fastest force field-based method that computes the free energy of binding, as compared to the other computational free energy methods, such as free energy perturbation (FEP) or thermodynamic integration (TI) methods. The MM/PBSA calculation was performed using g-mmpbsa software. The calculated binding free energies are shown in Table 3. The average overall binding free energy of the Mpro-16 and AAK1-17 complexes are − 39.776 (± 41.068) kJ/mol and − 171.265 (± 53.984) kJ/mol, respectively.

**Table 3:** Calculated binding free energies of tested compounds 16 and 17 [kJ/mol].

Complex	$\Delta G$(kJ/mol)	van der Waal energy	Electrostatic energy	Polar solvation energy	SASA energy
Mpro-16	− 39.776 (± 41.068)	− 58.763 (± 51.562)	− 12.993 (± 21.540)	39.232 (± 68.765)	− 7.252 (± 6.553)
AAK1-17	− 171.265 (± 53.984)	− 203.855 (± 25.396)	− 150.308 (± 69.756)	209.561 (± 29.494)	− 26.663 (± 0.712)

### ADME study

**Figure 23:**
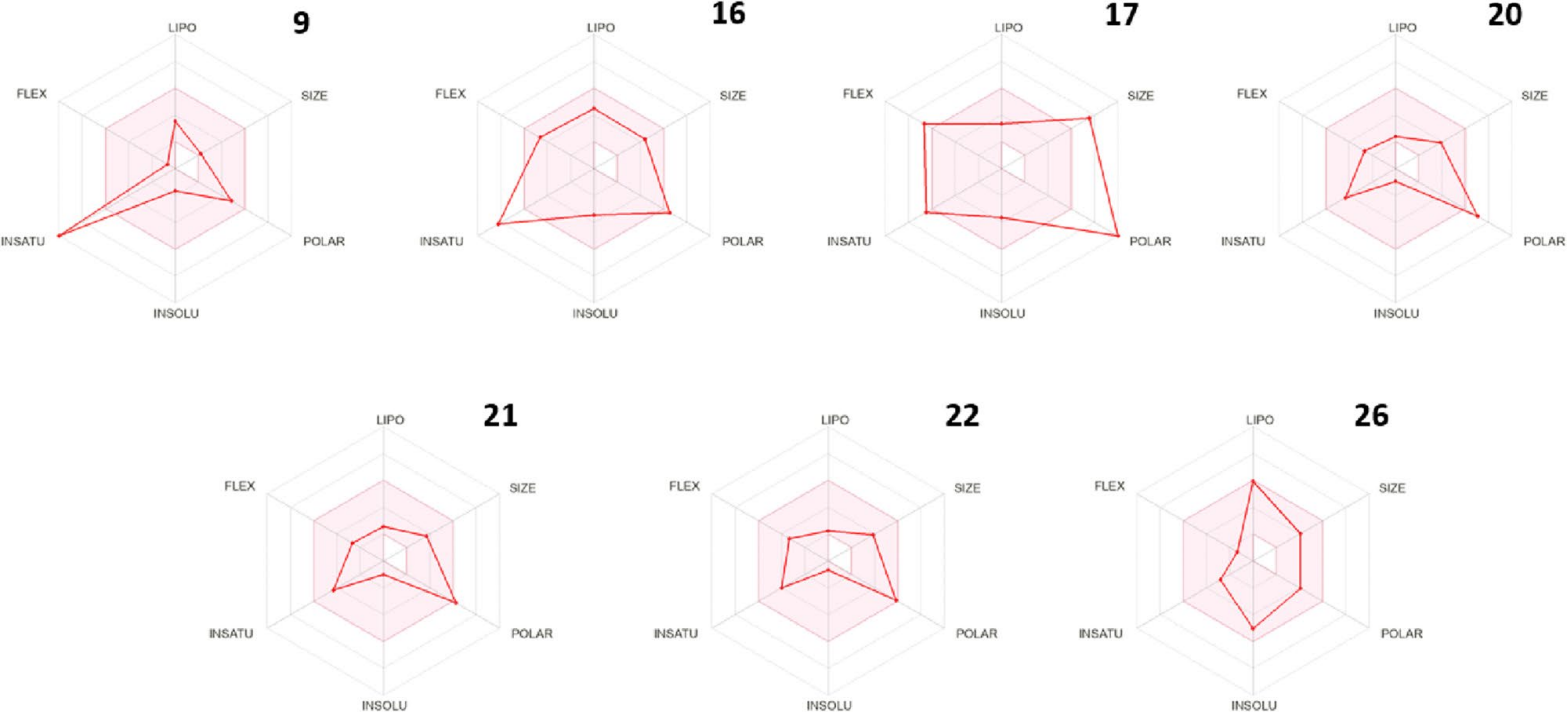
The bioavailability radar chart for the tested compounds (the colored zone is the suitable physicochemical space for oral bioavailability).

Selected compounds that showed good binding activity to both tested enzymes (**9**, **16**, **17**, **20**, **21**, **22** and **26**) were studied and tested through the SwissADME web tool. First, we studied the bioavailability through a radar chart that tests six parameters, Lipophilicity (LIPO), Size, Polarity (POLAR), Insolubility (INSOL), Unsaturation (UNSAT) and Flexibility (FLEX). Compounds **22** and **26** showed no violation, compounds **9**, **20** and **21** showed only one violation in the chart, while compounds **16** and **17** showed more than one violation due to their high hydrophilic characters of both compounds revealing their expected oral bioavailability (Fig. 23).

**Figure 24:**
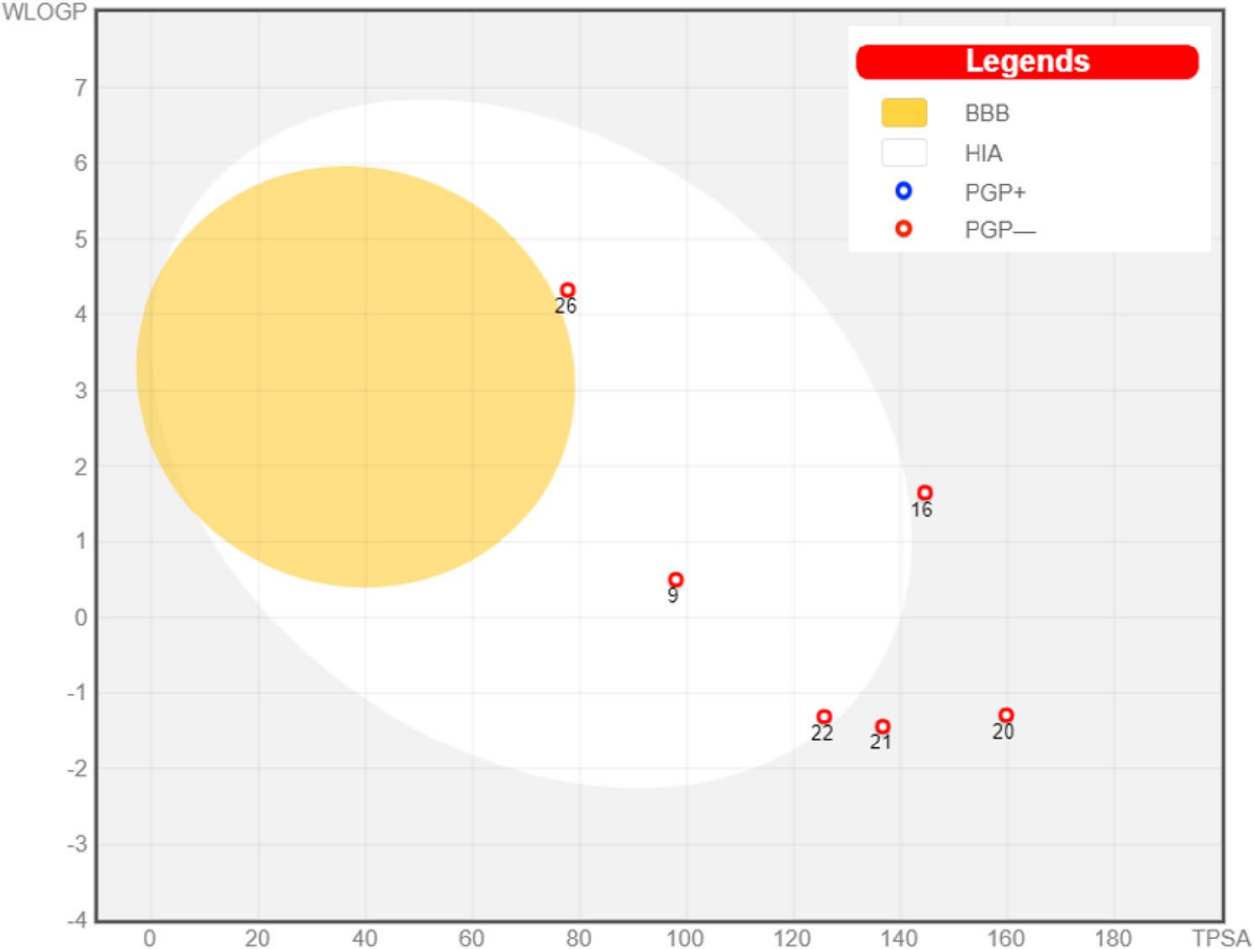
BOILED-EGG chart for the seven tested compounds (Yellow area is blood–brain barrier; BBB, white area is human intestinal absorption; HIA, red circles mean that these compounds are non-substrate for PGP).

The SwissADME server also provides a BOILED EGG chart to indicate the human intestinal absorption (white part), blood–brain barrier penetration (the yellow part)^44^, and the probability of the tested compound acting as a substrate for permeability glycoprotein (PGP) which is an efflux pump for many drugs (blue color if it’s a possible substrate or red color if it’s not)^45^. Only three of the tested compounds showed good gastrointestinal absorption (compounds **9**, **22** and **26**), which exhibited a good balance between lipophilic and hydrophilic characters. Other tested phenolic acid derivatives exhibited poor gastrointestinal absorption due to their high hydrophilic characters, especially for tannic acid (compound **17**), which did not appear in the chart due to extreme hydrophilic properties. All the seven tested compounds are not substrates for PGP, so they will not be susceptible to cell efflux (Fig. 24).

Eventually, applying Lipinski’s rule of five^46^ to predict the probability of the tested compounds being orally active, all showed no violations in the four parameters (log P, molecular weight, number of H-bond donor groups and number of H-bond acceptor groups) except for compound **17** (three violations) and compound **20** (one violation), this was because compound **17** (tannic acid) is extremely hydrophilic with high molecular weight (Table 4).

**Table 4:** Drug-likeness of the tested compounds (Lipinski’s rule of five).

Compound no.	Properties	Comment
**9**	Log P = 0.21 (< 5) Molecular weight = 170.12 g/mol (< 500) No of H-bond donor groups (OHs + NHs) = 4 (≤ 5) No of H-bond acceptor atoms (Os + Ns) = 5 (≤ 10)	No violation
**16**	Log P = 1.52 (< 5) Molecular weight = 360.31 g/mol (< 500) No of H-bond donor groups (OHs + NHs) = 5 (≤ 5) No of H-bond acceptor atoms (Os + Ns) = 8 (≤ 10)	No violation
**17**	Log P = -0.48 (< 5) Molecular weight = 636.47 g/mol (> 500) No of H-bond donor groups (OHs + NHs) = 11 (> 5) No of H-bond acceptor atoms (Os + Ns) = 18 (> 10)	Three violations
**20**	Log P = − 1.07 (< 5) Molecular weight = 315.25 g/mol (< 500) No of H-bond donor groups (OHs + NHs) = 6 (> 5) No of H-bond acceptor atoms (Os + Ns) = 9 (≤ 10)	One violation
**21**	Log P = − 0.91 (< 5) Molecular weight = 300.26 g/mol (< 500) No of H-bond donor groups (OHs + NHs) = 5 (≤ 5) No of H-bond acceptor atoms (Os + Ns) = 8 (≤ 10)	No violation
**22**	Log P = − 0.81 (< 5) Molecular weight = 314.29 g/mol (< 500) No of H-bond donor groups (OHs + NHs) = 4 (≤ 5) No of H-bond acceptor atoms (Os + Ns) = 8 (≤ 10)	No violation
**26**	Log P = 3.82 (< 5) Molecular weight = 332.43 g/mol (< 500) No of H-bond donor groups (OHs + NHs) = 3 (≤ 5) No of H-bond acceptor atoms (Os + Ns) = 4 (≤ 10)	No violation

The overall data shows that compounds 16 (rosmarinic acid) and 17 (tannic acid) require structural modifications to improve their pharmacokinetic properties and to be suitable for oral administration. Toxicity profile of our most promising natural acids 16 and 17 against Mpro and was investigated through lazar toxicity predictor, the obtained results revealed that both (16) rosmarinic acid and (17) tannic acid are non-carcinogenic in mice, rats and rodents model, additionally, AAK1 showed to be non-mutagenic in *Salmonella typhimurium* model. With Maximum Recommended Daily Dose in Human equal 4.35 (mg/kg_bw/day) and 137.0 (mg/kg_bw/day) respectively.

## Conclusion

The objective of this study was to find natural phenolic acids that can target SARS-CoV-2's main protease (Mpro) and adaptor-associated protein kinase 1 (AAK1) enzymes. The researchers employed several molecular modelling techniques such as pharmacophore mapping, molecular docking, molecular dynamics, and ADME studies. Rosmarinic acid (**16**) and 2,5-dihydroxybenzoic-5-O-glucoside (**20**) demonstrated remarkable binding affinities for both target enzymes and, as such, could act as dual inhibitors. Tannic acid (**17**) showed good potential in fitting all the required pharmacophoric features in AAK1 with the lowest docking score. These results were confirmed through pharmacophore mapping, molecular docking, and dynamic studies over 50 ns and extended to 100 ns. In terms of the ADME study, Rosmarinic acid has low bioavailability due to its high polarity and thus requires structural modification to improve its bioavailability as a promising dual target for COVID-19 prevention via main protease inhibition and endocytosis inhibition.

## Data Availability

All data generated or analyzed during this study are included in this article.
